# The metabolic fate of oxaliplatin in the biological milieu investigated during *in vivo* lung perfusion using a unique miniaturized sampling approach based on solid-phase microextraction coupled with liquid chromatography-mass spectrometry

**DOI:** 10.3389/fcell.2022.928152

**Published:** 2022-08-25

**Authors:** Mariola Olkowicz, Hernando Rosales-Solano, Khaled Ramadan, Aizhou Wang, Marcelo Cypel, Janusz Pawliszyn

**Affiliations:** ^1^ Department of Chemistry, University of Waterloo, Waterloo, ON, Canada; ^2^ Latner Thoracic Surgery Research Laboratories, Toronto General Hospital Research Institute, University Health Network, Toronto, ON, Canada; ^3^ Division of Thoracic Surgery, Department of Surgery, University Health Network, University of Toronto, Toronto Lung Transplant Program, Toronto, ON, Canada

**Keywords:** colorectal cancer, pulmonary metastases, adjuvant chemotherapy, in vivo lung perfusion, oxaliplatin, solid phase microextraction, spatial and temporal drug mapping, metabolomics

## Abstract

Adjuvant chemotherapy after pulmonary metastasectomy for colorectal cancer may reduce recurrence and improve survival rates; however, the benefits of this treatment are limited by the significant side effects that accompany it. The development of a novel *in vivo* lung perfusion (IVLP) platform would permit the localized delivery of high doses of chemotherapeutic drugs to target residual micrometastatic disease. Nonetheless, it is critical to continuously monitor the levels of such drugs during IVLP administration, as lung injury can occur if tissue concentrations are not maintained within the therapeutic window. This paper presents a simple chemical-biopsy approach based on sampling with a small nitinol wire coated with a sorbent of biocompatible morphology and evaluates its applicability for the near-real-time *in vivo* determination of oxaliplatin (OxPt) in a 72-h porcine IVLP survival model. To this end, the pigs underwent a 3-h left lung IVLP with 3 doses of the tested drug (5, 7.5, and 40 mg/L), which were administered to the perfusion circuit reservoir as a bolus after a full perfusion flow had been established. Along with OxPt levels, the biocompatible solid-phase microextraction (SPME) probes were employed to profile other low-molecular-weight compounds to provide spatial and temporal information about the toxicity of chemotherapy or lung injury. The resultant measurements revealed a rather heterogeneous distribution of OxPt (over the course of IVLP) in the two sampled sections of the lung. In most cases, the OxPt concentration in the lung tissue peaked during the second hour of IVLP, with this trend being more evident in the upper section. In turn, OxPt in supernatant samples represented ∼25% of the entire drug after the first hour of perfusion, which may be attributable to the binding of OxPt to albumin, its sequestration into erythrocytes, or its rapid nonenzymatic biotransformation. Additionally, the Bio-SPME probes also facilitated the extraction of various endogenous molecules for the purpose of screening biochemical pathways affected during IVLP (i.e., lipid and amino acid metabolism, steroidogenesis, or purine metabolism). Overall, the results of this study demonstrate that the minimally invasive SPME-based sampling approach presented in this work can serve as (pre)clinical and precise bedside medical tool.

## Introduction

Oxaliplatin (OxPt) is a third-generation platinum derivative that is currently used in various poly-chemotherapy schemes to treat advanced colorectal cancer (Di Francesco et al., 2002). When administered in combination with fluorouracil and leucovorin, or together with folinic acid and fluorouracil (FOLFOX), OxPt provides potent antitumor activity, presumably through the mechanism of blocking DNA replication and transcription. However, the benefits of OxPt-based treatments are limited by several recognized adverse effects, such as peripheral neuropathy, hematologic toxicity, and hypersensitivity effects (including severe anaphylaxis), as well as the development of resistance in the tumour. As such, there is significant interest in developing new strategies that would improve the tolerability and efficacy of platinum-based therapies (Di Francesco et al., 2002; Martinez-Balibrea et al., 2015; Branca et al., 2021). Over the last few decades, researchers have become increasingly interested in the use of platinum (IV) complexes, which are more kinetically inert than their platinum (II) counterparts and are characterized by lower reactivity towards biomolecules (Schueffl et al., 2021). Satraplatin has been the most thoroughly studied platinum (IV) pro-drug, having been tested in a clinical phase III trial for use in treating metastatic prostate cancer (Olszewski and Hamilton, 2010). While the findings showed that satraplatin significantly influenced progression-free survival, they also revealed that it was unable to achieve the primary endpoint of overall survival, which ultimately resulted in the denial of regulatory approval. This result was partially attributed to a lack of sufficient tumour specificity due to the premature reduction/activation of the drug in systemic circulation (e.g. in the red blood cells (RBCs)). Further attempts have been made to improve the tumour-targeting properties of platinum-based pro-drugs (Schueffl et al., 2021). To this end, researchers have proposed new albumin-targeted pro-drugs that demonstrate enhanced accumulation in malignant tissues, with the resultant overall increase in the concentration of intact drug in the cancer cells potentially serving to enhance the efficacy of OxPt therapy. Nonetheless, such approaches are still hampered by limitations associated with difficulties in reaching therapeutic levels within cancerous cells or common side effects when drug levels fall outside the therapeutic window (Li et al., 2019). Adding to the above, the lungs are the most frequent sites of extra-abdominal metastasis in patients with colorectal cancer. Thus, the development of new platforms that permit high doses of OxPt to be delivered locally to specific target organs, combined with analytical tools that enable precise drug level monitoring to ensure that tissue concentrations fall within the therapeutic window, are critical for effective cancer management.

Dr. Marcelo Cypel’s research group has recently developed a technique for isolated *in vivo* lung perfusion (IVLP) that facilitates the localized delivery of high doses of antineoplastic drugs to the lungs during surgical resection with the aim of preventing metastasis recurrence (dos Santos et al., 2014; Reck dos Santos et al., 2016). The IVLP uses the perfusion principles of the *ex vivo* lung perfusion (EVLP) platform (identical circuit and guidelines for perfusion), which was developed for the assessment and treatment of injured donor lungs prior to transplantation (Cypel et al., 2011). The optimized IVLP platform has already been used to administer sarcoma-based chemotherapy, specifically doxorubicin, (within 3 h-isolated left lung perfusion), thus demonstrating that high doses of therapeutic drugs can be safely administered without causing lung injury or systemic toxicity (Bojko et al., 2021). Currently, a phase I clinical trial is being conducted at University Health Network (UHN), with 9 patients having undergone surgery and adjuvant therapy *via* IVLP to date.

Solid-phase microextraction (SPME) has emerged as a novel, miniaturized sample-preparation approach that has been shown to be useful for the extraction/analysis of a broad range of metabolites and lipid species in a variety of matrices (Reyes-Garcés et al., 2018; Reyes-Garcés and Gionfriddo, 2019). The SPME procedure entails the insertion of an acupuncture-needle-sized microprobe (200 µm in diameter) coated with a biocompatible polymeric extraction phase (40 µm thickness) into tissue to the full length of the coating, followed by a short extraction period determined based on the compounds of interest. Since this approach does not require the removal of any of the tissue sample, it allows for repeated extractions/measurements, which is not be feasible with conventional biopsy. The simple design of coated SPME devices—which utilizes tuneable extraction phases and dimensions, while also providing negligible depletion—not only facilitates *in vivo* sampling, but it also enables the integration of sample collection and preparation into a single step (Ouyang et al., 2011; Reyes-Garcés et al., 2018; Reyes-Garcés and Gionfriddo, 2019). In addition, SPME is a solvent-less extraction technique that is based on equilibrium between the sample and the coating on the probe, which endows it with several advantages over traditional extraction methods, including simplicity, rapidity, and cleanliness. However, perhaps the most important feature of SPME is that it is sensitive enough to capture elusive pools of metabolites that are prone to rapid and extensive conversions, thereby allowing it to capture dynamic biochemical processes in the investigated system. Furthermore, coupling SPME with highly sensitive mass detectors permits xenobiotics (such as therapeutic drugs) tracking and the profiling of specific metabolic pathways or global metabolites, thus providing a snapshot of the entire metabolome in “one go.”

The main advantages of biocompatible SPME probes are: their suitability for direct exposure to complex biological matrices without a prior sample pre-treatment step; their high selectivity for small molecules; and, most importantly, their suitability for *in vivo* and non-destructive sampling (Ouyang et al., 2011; Piri-Moghadam et al., 2017; Reyes-Garcés et al., 2018). SPME’s applicability for *in vivo* tissue sampling has been demonstrated in numerous pre-clinical and clinical studies involving the sampling of the brain, liver, lungs, and heart (Reyes-Garcés and Gionfriddo, 2019). For instance, Lendor et al. (2019) applied an interesting miniaturized SPME device (total probe diameter: 195 µm) with multisite measurement capabilities for the extraction of multiple neurotransmitters within a single sampling event in a macaque brain (Lendor et al., 2019). With regard to targeted metabolite profiling in different brain areas, SPME microprobes have been also successfully applied for the in-depth profiling of up to 52 oxylipins in the brains of conscious moving rats (Napylov et al., 2020). Furthermore, recent studies have demonstrated that not only can SPME facilitate the *in vivo* extraction of a narrow metabolite/lipid subset from the brains of freely moving animals, but it can also be applied to concomitantly monitor changes in multiple metabolite or lipid classes to provide novel information about the biochemical pathways affected by a given treatment (i.e., deep brain stimulation or fluoxetine administration) (Reyes-Garcés et al., 2019; Boyaci et al., 2020). Nonetheless, the most compelling application of SPME for *in vivo* tissue analysis is for non-destructive organ (liver, heart, or lung) sampling to support decision making prior to transplantation, or for isolated lung sampling during chemo-perfusion to monitor spatial and temporal drug biodistribution or to identify potential markers associated with drug activity (Bojko et al., 2014; Bojko et al., 2021). In this context, Bojko et al. employed C8/SCX devices to perform spatial and temporal resolution mapping of the lung during local high-dose doxorubicin delivery *via* IVLP (Bojko et al., 2021).

In this paper, we propose a biocompatible SPME technology as a minimally invasive analytical strategy to assist in the near-real-time measurement of OxPt (and its metabolites) in tissue and perfusate during a porcine IVLP 3-days survival study. Additionally, a preclinical model is also investigated to further demonstrate the proposed technique’s applicability for monitoring changes/alterations in metabolomic patterns throughout IVLP. Finally, comprehensive time-course metabolite profiling is conducted, and dysregulated biochemical pathways are identified in an attempt to develop a deeper understanding of the manifold processes that occur in perfused lungs, thus enabling improvements in targeted treatments.

## Materials and methods

### Chemicals and materials

OxPt and internal standard (IS)—namely, carboplatin, diaquo-DACH platinum, and dichloro-DACH platinum (transient reactive species formed during nonenzymatic OxPt biotransformation)—were purchased from Toronto Research Chemicals Inc. (North York, ON, Canada). Stock solutions (2 mg/ml) containing OxPt, its metabolites, or carboplatin were prepared by dissolving an appropriate amount of the relevant compound in DMSO, followed by storage at −80°C until further use. The relevant OxPt standard working solutions (which were further used to prepare the calibration standards and quality control samples) were prepared by serially diluting the OxPt stock solution with water.

The SPME fibres used in this study consisted of a 200 µm nitinol wire with an extraction phase length of 8 mm and a coating thickness of 40 μm, with mixed-mode (MM) particles, which are a combination of benzenesulfonic acid (strongly acidic cation exchanger) and octyl functionalized silicate (SCX/C8), being selected as the extraction phase. Additionally, hydrophilic-lipophilic balanced (HLB) particles and C18 coating were investigated alongside the C8-SCX coating in the early optimization phase to determine the optimal conditions for the extraction/enrichment of the chemotherapeutic drug and its catabolites. The MM and C18 fibres (40 μm thickness, 5 μm particle size) were kindly provided by Millipore Sigma (Bellefonte, PA, United States), while the HLB probes (40 μm coating thickness) were manufactured in-house by repeatedly dipping nitinol wires in a slurry containing polyacrylonitrile (PAN) as a binding agent dissolved in dimethylformamide (DMF) and 5 μm HLB particles.

### SPME method development

#### SPME protocol

The extraction protocol was optimized using phosphate buffered saline (PBS) solution (for perfusate samples) and homogenized lamb’s lungs (for tissue samples), which served as a surrogate matrix. Prior to use (at the time of condition optimization and during lung sampling in the hospital facility), the fibres were sterilized for 45 min *via* steam autoclaving at 121°C in a Market Forge Sterilmatic Sterilizer (model STM-E type C; Middleby Corp., Elgin, IL, United States). Next, the sterile fibres were placed in an MeOH/H_2_O solution (1:1, v/v) to condition the extraction phase. After the conditioning step, the fibres were used to perform extractions under static conditions in either 300 µL of PBS solution or 10 g of homogenized lamb’s lung spiked with OxPt. The probes were subsequently wiped with a Kimwipe (Millipore Sigma, Burlington, MA, United States) to remove any loosely attached cell components and rinsed manually in water for 5 s. Finally, the extracts for LC-MS analysis were obtained by desorbing the fibres in an organic-aqueous solvent for 60 min at 1,500 rpm agitation.

### Investigation of performance of SPME coatings containing different functionalities

8-mm MM, C18, and HLB fibres were exposed to PBS solution spiked with the OxPt at a concentration of 1–40 µg/ml (mg/L) for 20 min at room temperature and under static conditions. Three-six fibre replicates were used per coating chemistry for each concentration level. Following extraction, each fibre was wiped with a Kimwipe, quickly dipped in water, and the analytes were desorbed under constant vortex agitation (1,500 rpm) for 1 h.

### Extraction time profile

The extraction time profiles for OxPt were evaluated to determine the extraction kinetics, equilibration time, and optimal extraction time that would enable the preservation of an abundant fraction of the intact drug during *in vivo* SPME sampling from lung tissue or perfusate. Sterile MM fibres were employed to perform extractions from 10 g of homogenized lamb’s lung spiked with OxPt at a concentration of 20 μg/g. Extractions were performed in sextuplicate at intervals of 10, 20, 30, 60, and 120 min (two independent experiments in triplicate), while the extraction time profile in perfusate samples was determined by sampling PBS solution at 5, 10, 20, 30, and 60 min using an OxPt concentration of 20 µg/ml. The preconditioning of extractive phases, rinsing, and desorption steps were carried out using the above-described conditions.

### Red blood cell (RBC) partitioning/binding of the drug and the degree of drug-protein binding that may affect its efficiency

OxPt at 40 µg/ml was incubated in Steen Solution, raw perfusate (collected before drug administration), and porcine plasma at 37°C for 3 h. At relevant intervals (1, 2, and 3 h of incubation), the MM-SPME probes were used to perform extractions from the aliquots containing the incubated solution. After analyte recovery *via* desorption in an aqueous-organic solvent, the obtained extracts were subjected to LC/MS analysis. The PBS solutions spiked with OxPt were also utilized as controls.

### SPME sampling of the left lung and perfusion fluids throughout IVLP

**FIGURE 1:**
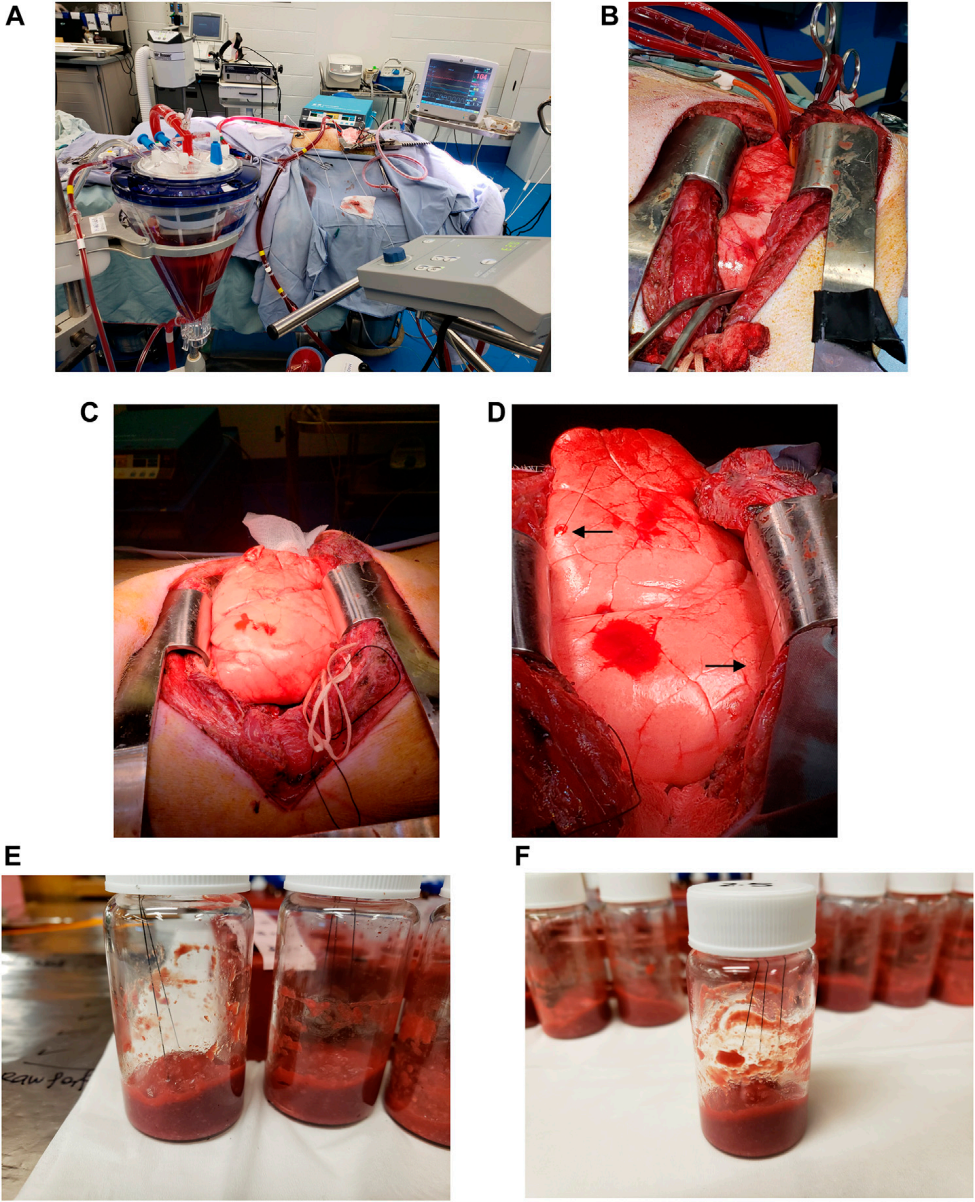
Bio-SPME sampling of porcine lungs during *in vivo* lung chemo-perfusion (IVLP). **(A-D)** Photographs of the IVLP circuit along with the solid-phase microextraction (SPME) probes applied for *in vivo* and non-destructive sampling of small-molecular-weight molecules, including oxaliplatin and its metabolites. As can be seen, the microprobes, which are approximately the size of an acupuncture needle (0.28 mm diameter), are inserted into upper lobe and lingula such that their entire 8 mm coating is fully immersed. **(E**,**F)**Preparation of matrix-matched calibration curve samples for accurate drug quantitation using homogenized lamb lung as a surrogate matrix.

Male Yorkshire pigs weighing an average of 40 ± 5 kg underwent a 3-h left lung IVLP procedure (Figure 1). OxPt (Pfizer, Kirkland, QC, Canada) was administered to the perfusion circuit reservoir directly as a bolus at doses of 5, 7.5, and 40 mg/L after full perfusion flow had been established. A detailed description of the left lung perfusion procedure, perfusion circuit, priming solution composition, and protective perfusion/ventilation strategy used in this study has been provided elsewhere (i.e., Ramadan et al., 2021). The experimental protocol was approved by the Institutional Review Board (IRB) at the University Health Network (UHN; Toronto, ON, Canada) and the University of Waterloo’s Research Ethics Board (# 40573).

For lung sampling, two MM-SPME fibres were placed in the upper and middle sections of the lung at predetermined time points over the course of the procedure (Figure 1), with sampling taking place prior to drug administration, hourly during IVLP, and once during blood reperfusion. The extraction of the drug/metabolites lasted for a duration of 20 min.

For the perfusate sampling, raw perfusate and supernatant (RBC free) were subjected to on-site extractions. The perfusate samples were collected just before the chemotherapeutic drug was injected into the perfusion circuit and again at 1, 2, and 3 h after administration. Perfusate sampling was conducted using 3 MM and three C18 fibres to perform a 20-min static extraction for each type of collected fluid.

After each SPME sampling had been completed, the fibres were wiped with a Kimwipe, rinsed manually in water for 5 s, and immediately placed in dry ice.

### LC-ESI-MS operating conditions

Before instrumental analysis, the MM-SPME fibres were desorbed in 60 µL of an ACN/H_2_O (8/2, v/v) mixture in glass vials with an insert and caps. To facilitate the desorption of analytes from the fibres to the surrounding solution mechanical agitation at 1,500 rpm was applied for 60 min.

Following desorption, targeted LC-MS/MS analyses were performed using a Dionex™ UltiMate 3000 UHPLC system (Thermo Fisher Scientific, Waltham, MA United States) interfaced *via* a heated electrospray (H-ESI) ion source to a TSQ Quantiva™ triple-stage quadrupole mass spectrometer (Thermo Fisher Scientific, Waltham, MA United States). The chromatographic separations of OxPt/its metabolites and the IS were performed on an Xbridge HILIC (Hydrophilic Interaction Liquid Chromatography) column (2.1 × 100 mm, 3.5 µm; Waters Corporation, Milford, MA, United States) using a two-solvent system (Solvent A: 50:50 IPA/H_2_O with 5 mM ammonium acetate; Solvent B: ACN). The refrigerated autosampler was set at 4°C, and the column heater was maintained at 30°C. The mobile phase flow was set to 0.400 ml/min, and the following gradient program was used: 0–1 min 95% B; 1–1.5 min 95–40% B; 1.5–3.5 min 40% B; 3.5–4 min 40–95% B; 4–7 min 95% B. An injection volume of 10 μL was used for all analyses. The effluent from the LC column was directed to the ion source of the mass spectrometer, which was operated in selected reaction monitoring (SRM) positive-ion mode. For the determination of OxPt, its metabolites, and the IS (carboplatin), the two most sensitive/specific ion transitions (one used for quantification and the other for confirmation) and the optimal collision energies (CE) were selected as follows: 1) oxaliplatin: 398.1 → 306.0/308.0 (CE: 25/20 V); 2) diaquo-DACH platinum: 386.2 → 354.0/306.0 (CE: 20/29 V); 3) dichloro-DACH platinum: 422.1 → 344.0/306.0 (CE: 22/36 V); and 4) carboplatin: 372.1 → 293.9/355.0 (CE: 17/10 V). The LC/MS system, data acquisition, and processing were managed using the Xcalibur software package (ver. 2.1., Thermo Scientific).

### Metabolomic and lipidomic investigations

The SPME extracts initially subjected to targeted drug/metabolites determinations were subsequently used for untargeted/global metabolomic determinations. To this end, relevant LC/MS analyses were performed using a Vanquish UHPLC system (Thermo Fisher Scientific, Waltham, MA United States) interfaced to a high-resolution benchtop Exactive Orbitrap mass spectrometer (Thermo Fisher Scientific, Waltham, MA United States). Data were collected in ESI+ (positive ion) and ESI− (negative ion) mode in two different analytical runs using conditions that have been detailed in a prior work (see Olkowicz et al., 2021). In order to monitor LC/MS performance across sample runs, a quality control (QC) sample was prepared as a pooled mixture of sample aliquots and injected along the sequence.

For the lipidomic investigations, the C18-SPME fibres were desorbed in 60 µL of MeOH/IPA/H_2_O (45:45:10, v/v/v) under mechanical agitation at 1,500 rpm for 60 min. Chromatographic separation was achieved with an XSelect CSH C18 column (2.1 × 75 mm, 3.5 µm; Waters Corporation, Milford, MA, United States) using a two-solvent system, which has been detailed elsewhere (i.e., Solvent A: 40:60 MeOH:H_2_O with 10 mM ammonium acetate and 1 mM acetic acid in positive mode, and 0.02% acetic acid in negative mode; Solvent B: 90:10 IPA:MeOH with 10 mM ammonium acetate and 1 mM acetic acid in positive mode, and 0.02% acetic acid in negative mode) (Olkowicz et al., 2021).

The LC/MS data were initially processed with ProteoWizard—which includes a very handy tool (msconvert) for converting raw data into mzXML format (Chambers et al., 2012)—and subsequently analyzed for peak extraction, grouping, retention-time correction, and peak filling using the XCMS software package (Huan et al., 2017). The XCMS parameters were optimized using the IPO package and adjusted to be slightly more inclusive (Libiseller et al., 2015; Albóniga et al., 2020). The xMSannotator Integrative Scoring Algorithm was employed to annotate the extracted peaks, with METLIN, KEGG, and LIPID MAPS being used as reference databases (Uppal et al., 2017). Only unique features with medium-to-high confidence matches annotated by METLIN/KEGG/LIPID MAPS were selected for further investigation. Unsupervised principal component analysis (PCA) was performed on log-transformed, mean-centred data to detect potential outliers, assess data quality, and visualize major structures in the data. PCA score plots were generated to show clusters of samples based on their similarity, while PCA loading plots were created to identify the components that contributed to positive separation among studied groups/conditions. Once the PCA score and loading plots had been constructed, supervised PLS-DA (partial least-squares discriminant analysis) was performed, followed by model validation and variable selection, the latter of which being achieved using a variable influence on projection (VIP) and an absolute value of p (corr) greater than 1.5 and 0.5, respectively. Any statistical treatment of data was carried out using the web-based MetaboAnalyst 5.0 software package (Chong et al., 2019).

### Data analysis for targeted determinations of OxPt

All SRM data were processed and visualized using the Xcalibur 2.1. Quan Browser software package (Thermo Scientific), while the quantification of OxPt was performed using the matrix-matched calibration method. Eleven-point calibration curves (including blank samples plus IS and triplicate determinations for each level) spanning a 1000-fold concentration range were constructed with linear regression analysis and 1/x (x = concentration) weighting. The covered ranges were 0.1–100 µg/ml (mg/L) and 0.1–100 µg/g for perfusion fluids and lung tissue, respectively. Data were presented as the mean ± SD for repeated determinations of analyte per sampling time point (perfusate).

## Results

### Assessing protocol feasibility

The optimization framework for early-stage design included evaluations of the following parameters: 1) the selectivity/performance of SPME coatings with various functionalities (C8/SCX, C18, or HLB particles); 2) optimal settings for the extraction of intact OxPt that closely simulate *in vivo* SPME extraction conditions; 3) the time-course of sample collection; 4) OxPt’s RBC and protein-binding behaviour; and 5) the optimal instrumental and data-acquisition conditions for capturing and identifying a broad range of metabolites, including OxPt and its metabolites.

**FIGURE 2:**
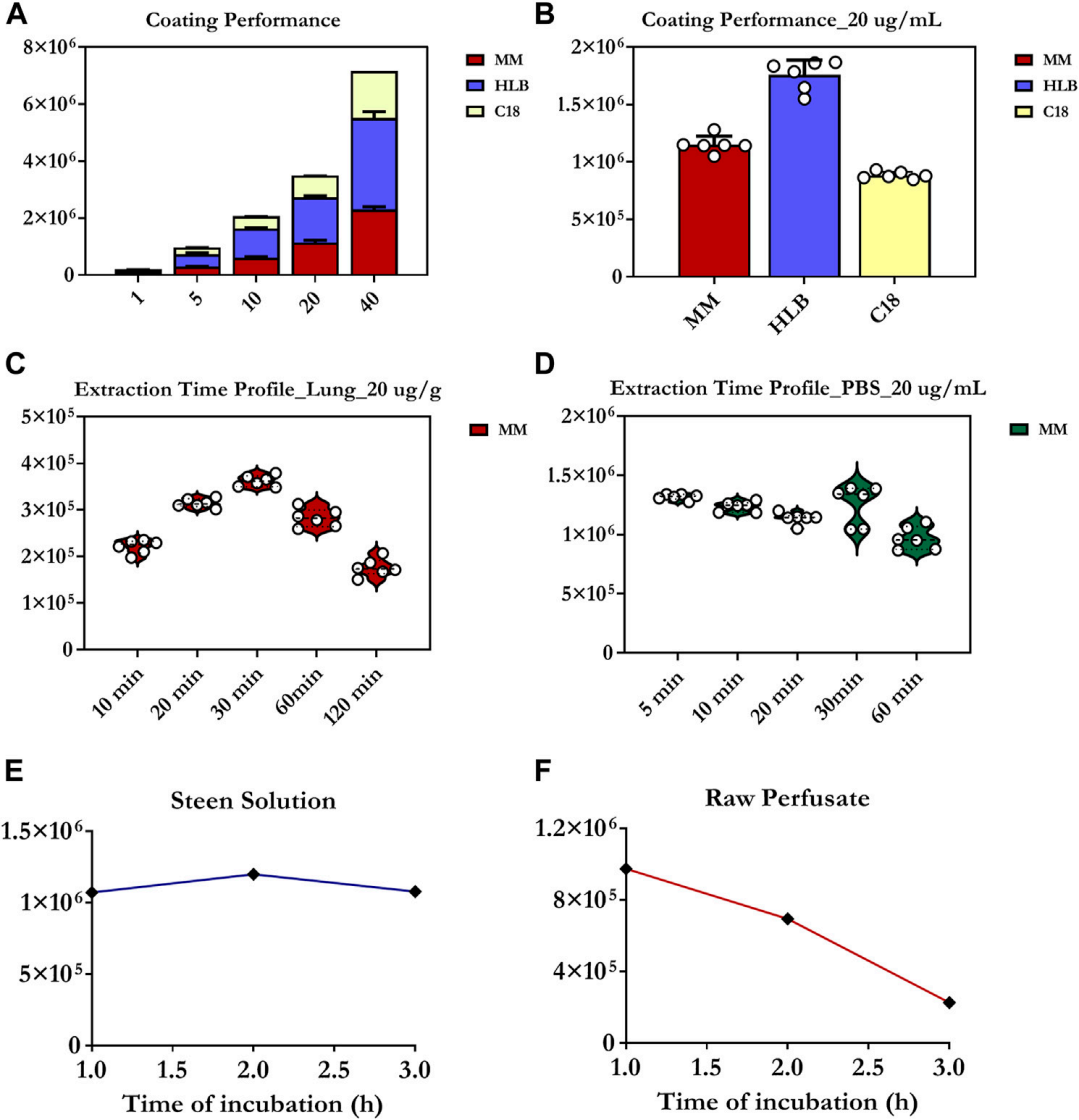
Evaluation of extractive phase selectivity/performance next to static extraction time profiles and degree of drug-protein/RBC binding. **(A,B)** Extraction performance of C8/benzenesulfonic acid (C8/SCX), C18, or HLB SPME coatings for the determination of oxaliplatin. **(C)** Extraction-time profile of oxaliplatin at 20 µg/g obtained from two different lamb lung samples (10 g of homogenized tissue). Each experimental point represents the median of 6 collection points (3 probes per lung per point). **(D)** Extraction-time profile of oxaliplatin at 20 µg/ml in phosphate buffered saline (PBS) (*n* = 6 for each time point). **(E,F)** Red blood cell (RBC) partitioning/binding of oxaliplatin and degree of drug-protein binding investigated at a concentration level of 40 µg/ml in Steen Solution and raw perfusate for a 3 h period. The values presented on *Y* axis correspond to chromatographic peak areas collected for oxaliplatin under particular studied conditions. In turn, each dot (in the graphs) represents fibre replicate. Three-six fibre replicates were used per coating chemistry for each concentration level.

Of the three tested coatings, the HLB coating provided the broadest metabolite coverage and greatest intensities for significant detected features, including OxPt and its catabolites (Figures 2A,B). The MM coating exhibited superior recovery for the majority of polar and moderately hydrophobic compounds compared to the C18 coating, and was therefore deemed more suitable for the analysis of hydrophilic platinum-based anticancer drugs in the lung. Since HLB fibres are not yet commercially available, thus potentially making their implementation in routine metabolomic workflows tedious/time-consuming, MM fibres are a good option, as they offer an acceptable compromise between suitability for high-throughput applications and balanced metabolite coverage, including for OxPt and its bio-transformative environment.

Next, static extraction at equilibrium conditions was proposed as the most reliable sampling strategy, as it can prevent matrix conditions from influencing SPME’s quantitation capabilities in the absence of a reference standard (added to the investigated matrix) (Figures 1E, F). As indicated in Figure 2C, 30 min was identified as the minimum extraction time required to achieve equilibrium extraction (in homogenized lung tissue) and, therefore, maximum sensitivity. Nonetheless, it is critical to note that, compared to static extraction, the insertion of fibres into a living system promotes faster extraction, presumably due to blood-flow-related convection, which means that equilibrium will always be reached faster during *in vivo* extraction (Roszkowska et al., 2018). As such, a sampling time of 20 min was selected for the final analyte (OxPt) determinations in the porcine lung models. In contrast, equilibrium appeared to be reached more quickly in the PBS solution using shorter extraction times (below 10 min) (Figure 2D). Overall, a sampling time of 20 min was selected for both cases (lung tissue, perfusate) to ensure that alterations in drug levels during chemotherapy could be monitored with high precision and reliability, thus providing a near-real-time profile of the biodistribution of OxPt in the lung tissue throughout IVLP next to its levels in the priming fluid.

Five sampling time points were also selected for investigation: 1) before OxPt administration, 2) at the first, second, and third hour during IVLP, and 3) during blood reperfusion. To minimize any possible organ stress associated with the insertion of the MM-SPME microprobes, the minimum number of fibres that would still yield sufficient biological information was used (i.e., two fibres in the upper and middle sections of the lung). In addition, the perfusate was sampled in parallel with lungs using 3 MM (OxPt and untargeted investigations) and three C18 (untargeted analysis) fibres for each sampling time point.

To study OxPt’s protein-binding properties with respect to albumin, and to measure its real-time concentration in the priming fluid, OxPt was incubated in Steen Solution at 37°C for 3 h. The maximum protein binding rate was found to be 55–60%, with equilibrium for drug-protein binding being reached at the first hour of incubation (Figure 2E). Conversely, when OxPt was incubated in raw perfusate, its uptake into the erythrocytes was rapid, with an average of 25–30% of the drug being partitioned into the erythrocytes over a 1 h-time period (Figure 2F).

Several column stationary phases, including pentafluorophenyl (PFP)-bonded phase, silica-based ZIC®-HILIC (zwitterionic) sorbent, and XBridge BEH HILIC, were tested for their ability to retain intact OxPt and its biotransformation products (Pt(DACH)Cl_2_, Pt(DACH)(OH)_2_). The results indicated that the Xbridge HILIC column with a dual-mode gradient elution program provided favourable retention and robust performance for polar solutes investigated in LC-SRM/MS mode (Supplementary Figures S1–S4).

In contrast, in untargeted metabolomic analysis, the objective is to separate as many compounds as possible in a single analytical run (Wishart, 2016; Azad and Shulaev, 2019; Jacob et al., 2019). Therefore, the stationary phase and chromatographic mode selected for such analyses should provide the broadest separation and selectivity for the widest possible range of metabolites. However, the required level of metabolite coverage may depend on the objectives of the study and it is generally accepted that no one analytical method is capable of comprehensively profiling the entire metabolome. In the current work, three stationary phases were tested with the aim of providing expanded metabolite coverage, namely: 1) PFP-bonded phase, 2) HILIC, and 3) the most common C18-bonded phase. Although the PFP and C18 phases offered similar reversed-phase (RP) selectivity, the PFP phase outperformed the C18 and HILIC phases in terms of the number of metabolic features retained, while the HILIC phase was able to retain the greatest proportion of highly polar metabolites. Ultimately, the PFP phase was deemed to provide the best performance and was selected for use in subsequent metabolomic investigations in the lung. Moreover, it is possible to increase metabolome coverage by utilizing data from both ionization modes, as the detection of particular metabolite/lipid species, such as acylcarnitines, fatty acids, and specific lipid classes, occurs in either positive or negative ionization mode. Furthermore, to capture perturbations in the hydrophobic fraction of compounds (throughout IVLP), C18-based microprobes were employed for perfusate sampling, with the extracts being analysed in RP-LC/MS mode.

### Tissue and perfusate OxPt levels

**FIGURE 3:**
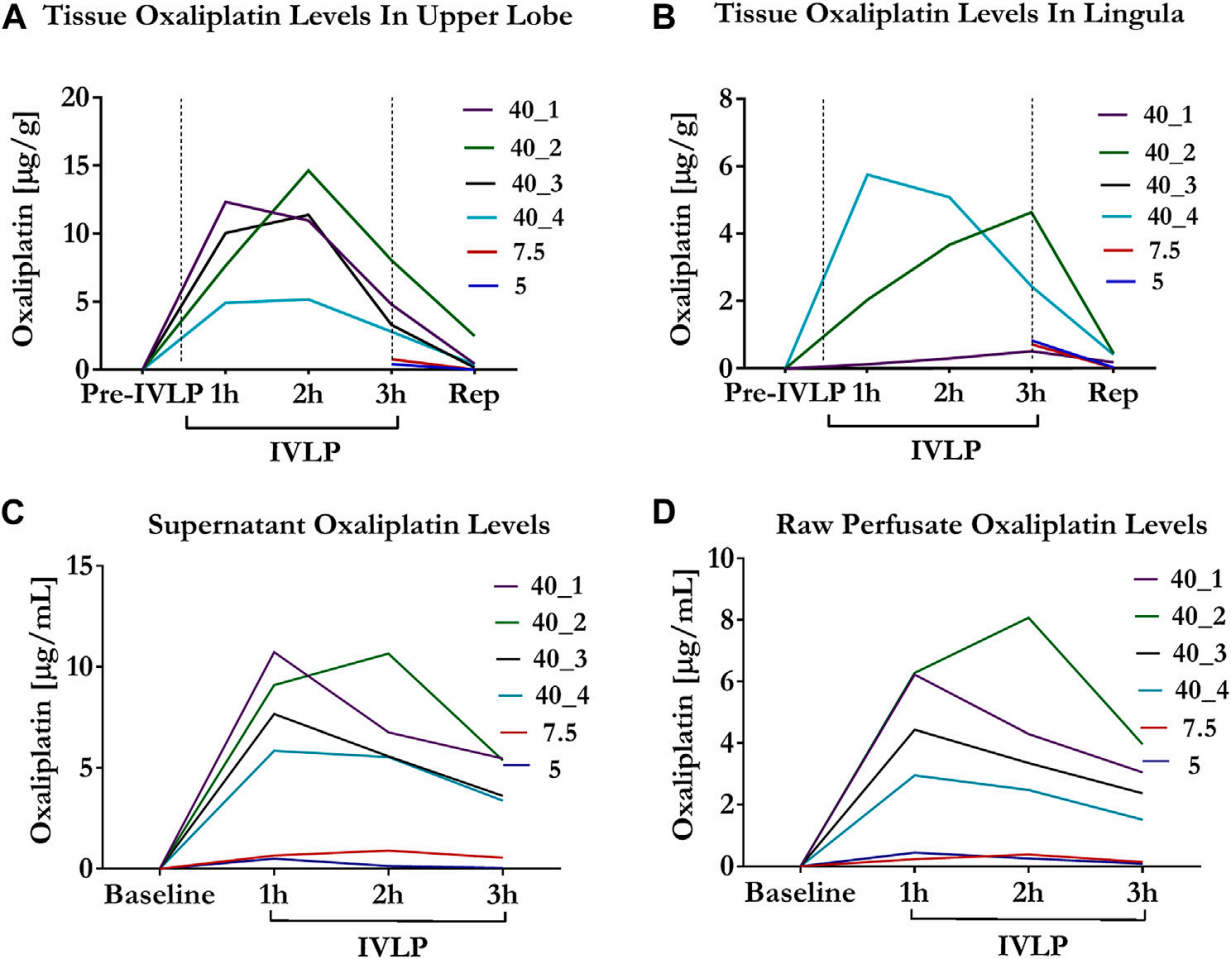
Oxaliplatin levels in tissue and perfusate during IVLP. **(A,B)** Tissue OxPt levels measured by Bio-SPME-LC/MS in upper lobe and lingula for three doses of the drug investigated (5, 7.5 and 40 mg/L). OxPt concentrations in the upper lobe presented a dose-dependent relationship, peaking at 2 h of IVLP (in most cases), whereas that trend was less evident in lingula. Notably, for cases treated with lower doses of OxPt (5 and 7.5 mg/L), samples were collected at the third hour of IVLP and during blood reperfusion. **(C,D)** Concentration of OxPt in perfusate measured by Bio-SPME-LC/MS during porcine IVLP. OxPt levels in perfusate exhibited a dose-dependent increase and peaked at 1 h of perfusion, with higher values noted in the supernatant vs. the raw perfusate.

Tissue measurements of OxPt concentrations revealed the rather heterogeneous distribution of the drug (over the course of IVLP) in different sections of the lung. This trend was most evident for higher doses, with higher concentrations of OxPt being noted (in most cases) in the upper section of the lung (Figures 3A,B). Furthermore, OxPt concentrations in the upper lobe appeared to have a dose-dependent relationship, peaking at 2 h of IVLP (in most cases) before rapidly declining to near-zero values at reperfusion. Conversely, drug levels in the lower lingula tended to be more inconsistent as the dosage increased; thus, the lingula might be not representative during sampling. Apart from the heterogenous biodistribution of OxPt in lung tissue, the findings also indicated relatively low tissue levels of the drug, which may be attributable, at least in part, to binding between OxPt and the albumin present in the Steen solution, its sequestration into erythrocytes, or its rapid and extensive nonenzymatic biotransformation.

Perfusate levels of OxPt peaked at the second hour of IVLP in animals being treated with lower doses of the drug (5, 7.5 mg/L) (Figure 3C). In contrast, the perfusate levels of OxPt peaked in animals being treated with higher doses at the first hour of IVLP, with concentrations subsequently declining over time. Furthermore, the OxPt in supernatant samples represented ∼25% of the entire drug 1 hour after injection.

Similar to the supernatant samples, raw perfusate levels of OxPt peaked at the second hour of IVLP in animals being treated with lower doses of the drug, whereas concentrations peaked at the first hour for animals being treated with a higher dose (40 mg/L) (Figure 3D). However, OxPt levels were lower in raw perfusate samples (vs. supernatant samples), thus confirming the tendency of the drug to bind/accumulate in RBCs.

OxPt levels during IVLP and reperfusion were also assessed in selected plasma samples. These results showed no detection of OxPt systemically, indicating an effective separation between the pulmonary and systemic circulations.

The proposed analytical method was developed for the determination of intact OxPt and two other cytotoxic platinum species that form during nonenzymatic OxPt conversion (diaquo-DACH platinum and dichloro-DACH platinum), which comprise the dominant metabolic routes of biotransformation. However, given the highly reactive nature of these species, it is likely that they are only present temporarily rather forming complexes with amino acids, proteins, DNA, and other macromolecules, as our determinations found only trace levels of these compounds (Supplementary Figures S1–S4).

### Cellular metabolic landscape of the chemotherapy milieu at spatial and temporal resolution

As previous studies have demonstrated, SPME is suitable for the extraction and profiling of a broad range of metabolites and lipid species in different biomatrices, including elusive (unstable) fraction of compounds (Reyes-Garcés et al., 2018; Reyes-Garcés and Gionfriddo, 2019). The Bio-SPME extracts that were initially used to evaluate OxPt (and its metabolites) in tissue and perfusate samples were also used for the screening of other low-molecular-weight compounds using an LC-HRAM (High Resolution Accurate Mass) system. It should be noted that the global analyte investigations only used samples collected from cases treated with a 40 mg/L dose of OxPt.

**FIGURE 4:**
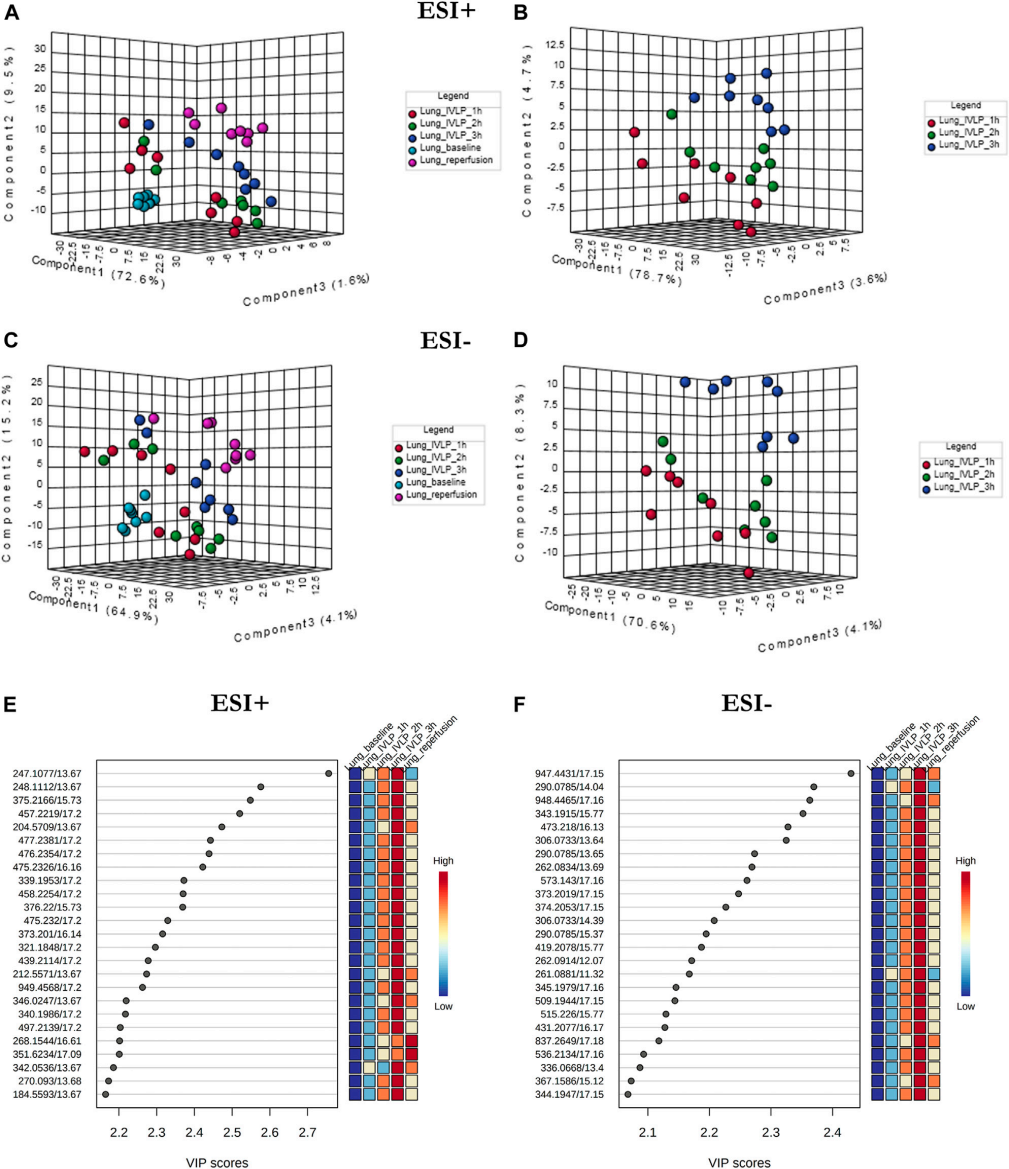
PLS-DA score plots of the metabolite profiles **(A–D)** for various sampling time-points during *in vivo* lung chemo-perfusion along with corresponding VIP values **(E,F)**. The presented score plots were created for metabolic features detected in **(A,B)** ESI+ and **(C,D)** ESI- PFP-based mode. The coloured dots represent individual samples that were collected during sampling of different sections of the lung. Light blue: lung sampling before drug administration. Red, green, dark blue: sampling at the first, second, and third hour of lung chemo-perfusion, respectively. Pink: sampling during blood reperfusion. A distinct separation of samples based on the time they were collected manifested clear metabolic differences in the lung metabolome in response to the applied therapy.

Figure 4 presents the PLS-DA results obtained in ESI+ (**A**, **B**) and ESI− (**C**, **D**) ionization modes for lung tissue samples obtained at different time points during the IVLP/surgical procedure. From the plots, it is evident that the samples collected at each time point generate relatively well-separated clusters, with separation appearing as a transitionary pattern from left to right in the plot. These results indicate that the proposed method provided clear discrimination among metabolomic or lipidomic patterns in the samples collected before commencing lung perfusion, during IVLP, and during blood reperfusion. Additionally, 148 (out of 958 detected) and 153 (out of 907 detected) metabolic features with a VIP score >1.5 were identified in ESI+ and ESI− modes, respectively. The top 25 discriminative features along with their corresponding VIP values are presented in Figures 4E,F. It is further important to note that most dysregulated compounds increased in abundance during IVLP.

**FIGURE 5:**
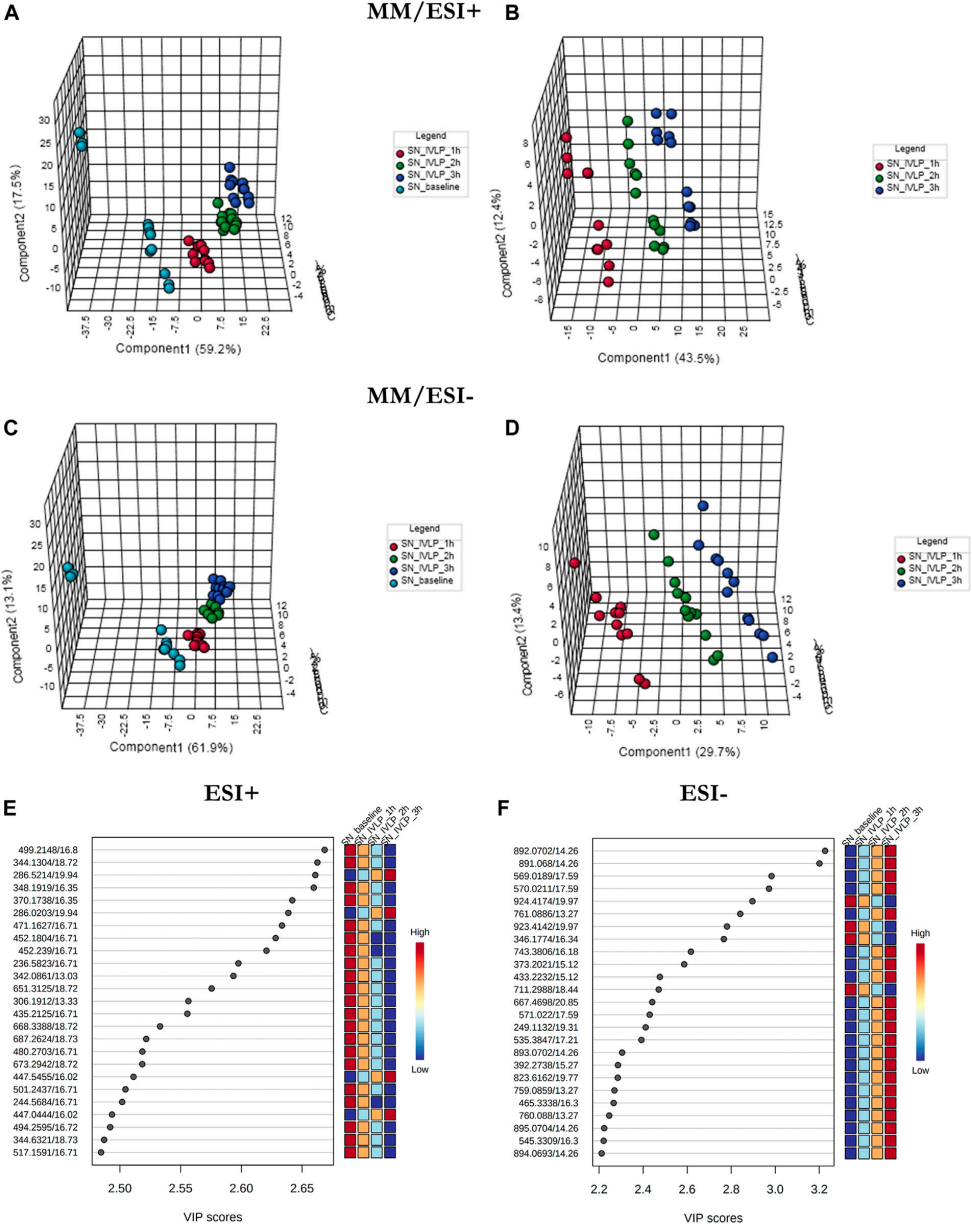
PLS-DA score plots (perfusate samples) comparing LC/MS metabolomic profiles among four **(A,C)** and three **(B,D)** conditions corresponding to relevant sampling time-points (pre-perfusion, and at the first, second, and third hour of IVLP). Excellent discrimination was observed among the studied conditions (pre-perfusion vs. IVLP) when the mixed-mode (MM) coating was used for extraction and PFP-based mode was used for analyte separation. SN baseline: supernatant samples collected at pre-perfusion. SN_IVLP_1h/2h/3h: samples collected at the first, second, and third hour of IVLP. **(E,F)** VIP schematic scores of PLS-DA analyses presenting the most discriminative metabolic variables.

**FIGURE 6:**
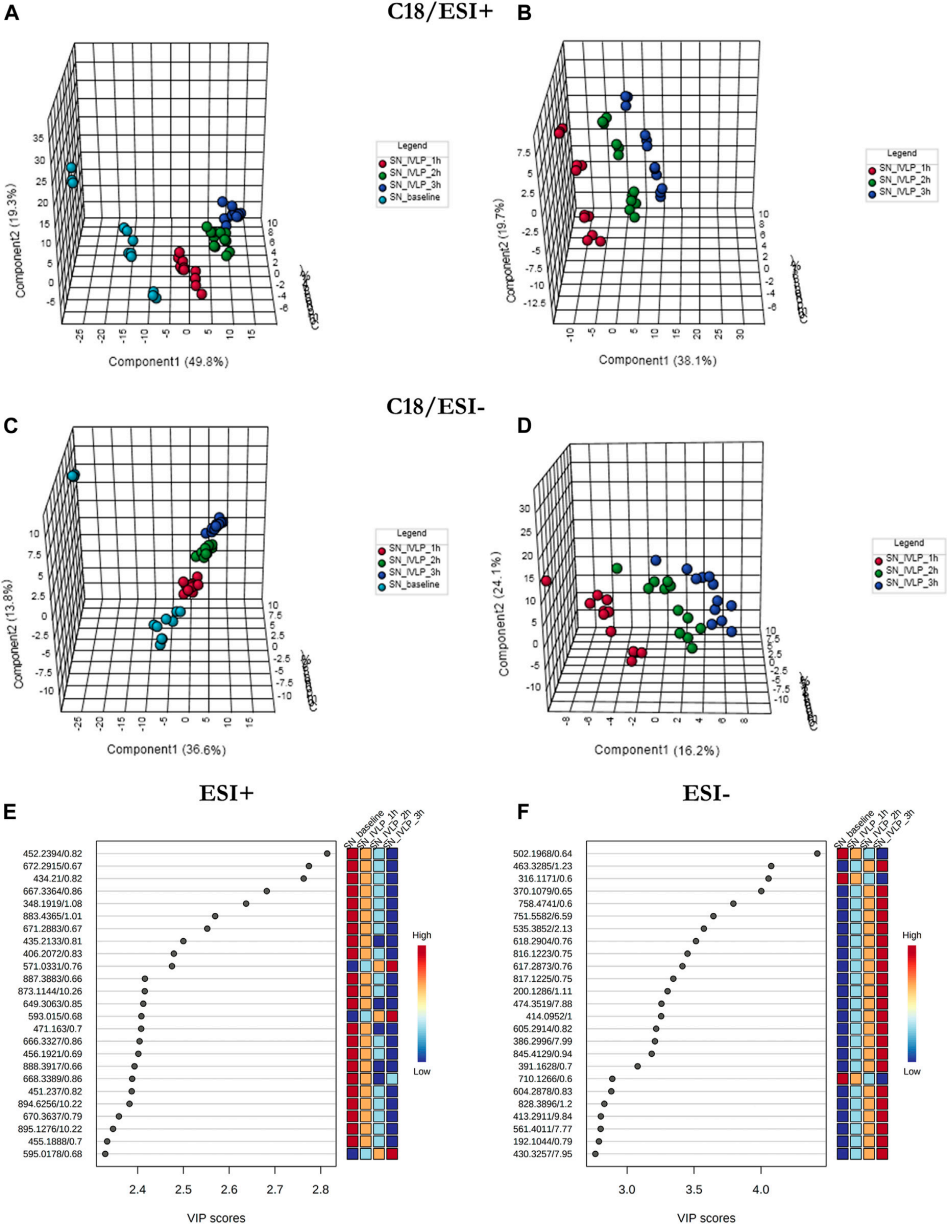
PLS-DA results for perfusate samples collected with C18-based microprobes. **(A-D)** The results show excellent discrimination of lipidomic profiles based on the time of sample collection. SN baseline: supernatant samples collected at pre-perfusion. SN_IVLP_1h/2h/3h: samples collected at the first, second, and third hour of IVLP. **(E**, **F)** VIP schematic scores of PLS-DA analyses presenting the most discriminative lipid species.

In addition to lung tissue, MM and C18 SPME microprobes were also used to perform extractions on perfusate samples. Specifically, metabolomic and lipidomic evaluations were performed on both the supernatant and raw perfusate samples. As can be seen in Figures 5A–D, 6A–D, the samples were clearly divided into 4/3 groups with highly distinct metabolic profiles coinciding with the time of sampling. Furthermore, VIP scores were calculated to determine the features responsible for the variance in the PLS-DA prediction models. In total, 133 variables detected in MM/ESI+ mode and 120 variables detected in MM/ESI–mode met the VIP score threshold (i.e., >1.5), as did 87 variables detected in C18/ESI+ mode and 133 detected in C18/ESI–mode. The top 25 dysregulated features (for a given analysis mode) are presented in Figures 5E,F, 6E, F.

Furthermore, the two-dimensional PCA score plots for the samples in both positive and negative ion modes revealed no outliers, with the tightly clustered QC samples (in relevant PLS-DA score plots) confirming detection stability and the high quality of the collected data (see Supplementary Figures S5–S11).

**FIGURE 7:**
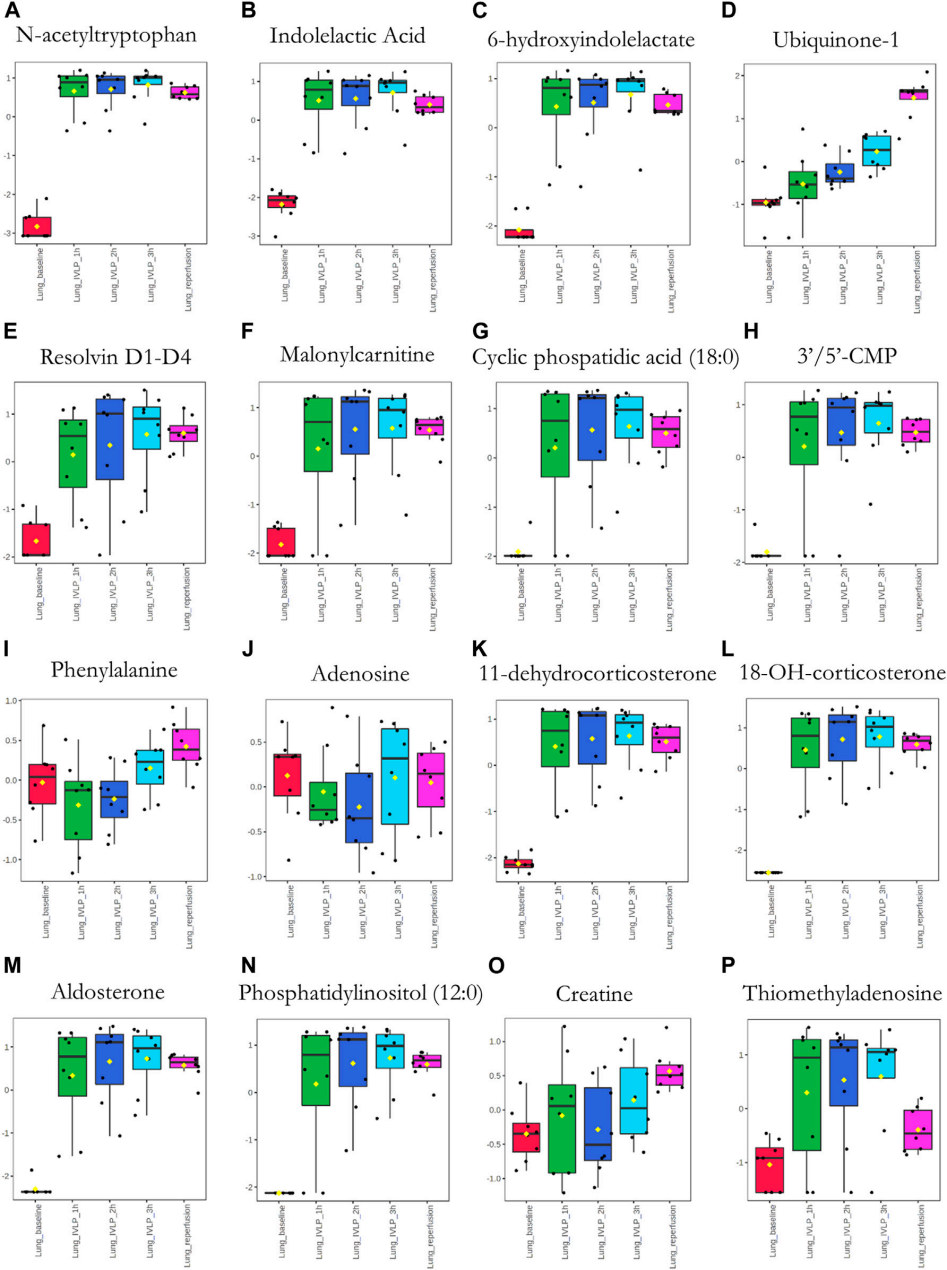
Box plots showing relative abundances of the top discriminatory metabolites or lipid species at different sampling points during the IVLP. The boxes denote interquartile ranges, the horizontal line inside each box denotes the median, and the top and bottom boundaries of the boxes represent the 25th and 75th percentiles, respectively. Each black dot presents a different sample. Lung_baseline: samples collected at pre-perfusion. Lung_IVLP_1h/2h/3h: samples collected at the first, second, and third hour of IVLP. Lung_reperfusion: samples collected 30 min post reperfusion.

**FIGURE 8:**
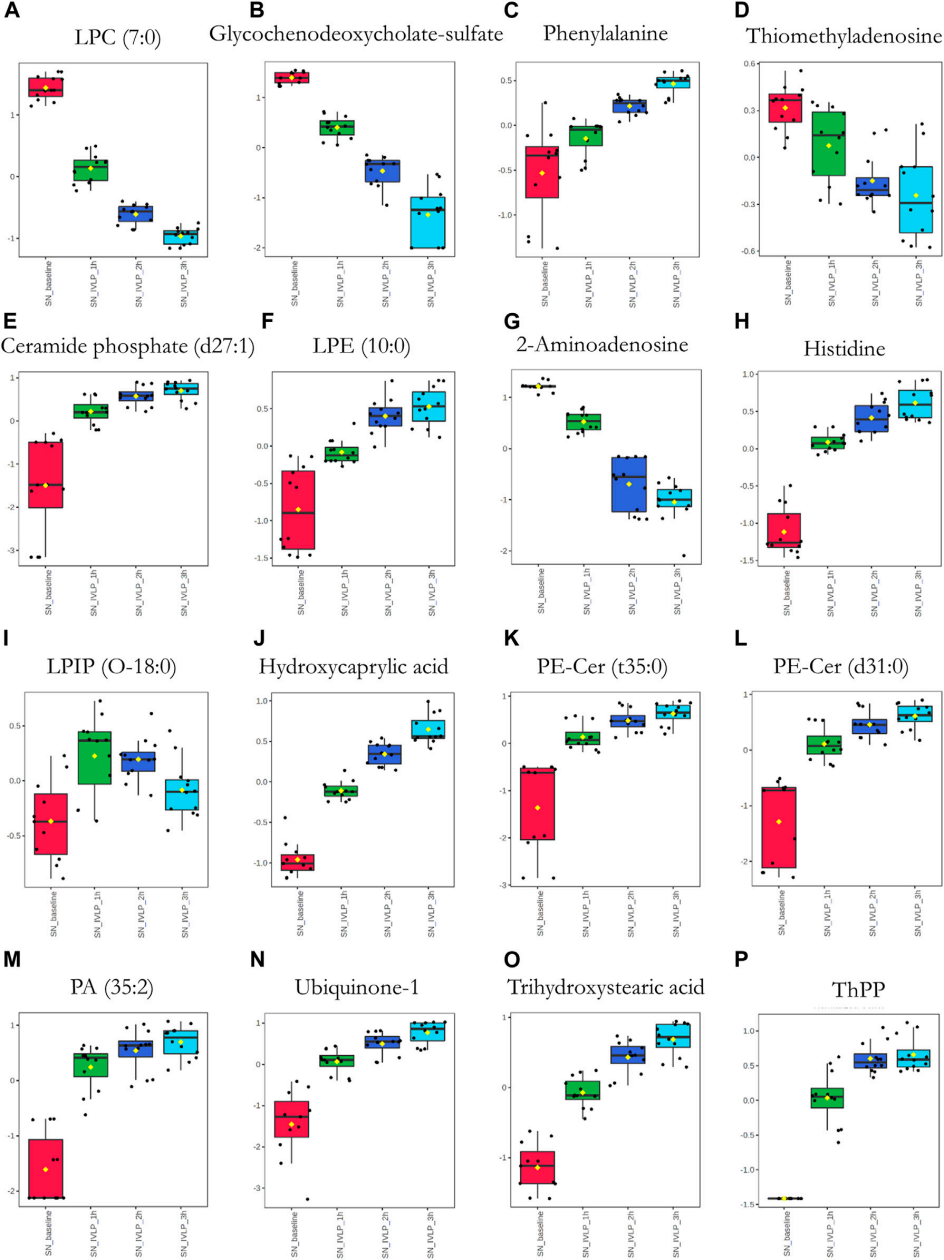
Box plots of metabolic features identified in the perfusate *via* MM probes showing the strongest differences between samples obtained at different points in the IVLP procedure. SN_baseline: samples collected before OxPt administration. SN_IVLP_1h/2h/3h: samples collected at the first, second, and third hour of IVLP.

**FIGURE 9:**
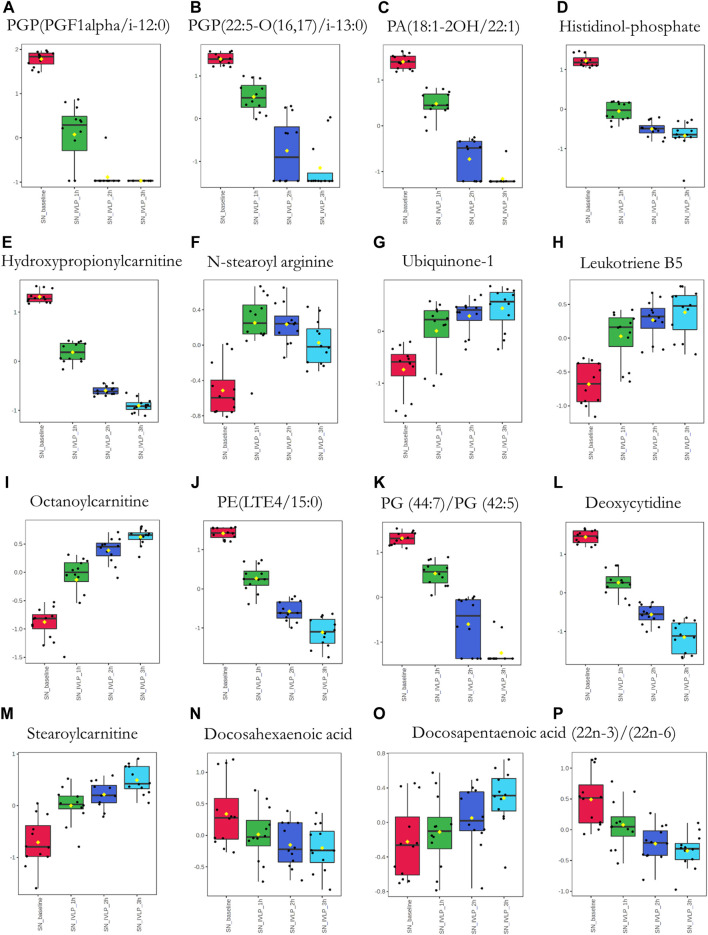
Box plots of metabolic features identified in the perfusate *via* C18 probes showing the strongest differences between samples taken at different points in the IVLP procedure. SN_baseline: samples collected before OxPt administration. SN_IVLP_1h/2h/3h: samples collected at the first, second, and third hour of IVLP.

Next, significant features were annotated based on the Metabolomics Standards Initiative guidelines (Sumner et al., 2007) and the International Lipid Classification and Nomenclature Committee recommendations (Liebisch et al., 2013). The putative annotations, which were based upon accurate mass database matches and tandem MS fragmentation data (where the latter were available), are presented in Supplementary Tables S1, S2 in the Supplementary Information. Additionally, box plots comparing the abundances of the annotated compounds over the course of IVLP are shown in Figures 7–9. Further details of the putative compound annotations and their extracted-ion chromatograms (XIC) can be found in the (Supplementary Tables S1–S2 and Supplementary Figures S12–S14).

Finally, we sought to determine which biochemical pathways were affected by the administration of chemotherapy. These results indicated that the application of chemotherapy disrupted pathways related to lipid (specifically, free fatty acid) and amino acid metabolism, such as the tryptophan/kynurenine, histidine, and phenylalanine pathways. Other candidate pathways potentially related to lung toxicity or injury include accelerated oxylipin generation, amplified steroidogenesis, and the production of reactive oxygen species (ROS). Moreover, profound alterations were observed in the metabolism of purine and pyrimidine, as well as the cellular lipid mediators. Thus, these results establish Bio-SPME as the perfect tool for detecting alterations to the cellular lipidome in response to chemotherapy-induced stress. Despite these encouraging results, further research will be required to determine whether the membrane lipid remodelling was a primary cellular adaptation to detrimental environmental conditions (Supplementary Tables S1–S2 and Figures 7–9).

## Discussion

OxPt is a third-generation platinum-based anticancer drug that is highly effective in preventing the growth of colorectal cancer and other malignancies, including lung, gastric, ovarian, and prostate cancer (Di Francesco et al., 2002; Martinez-Balibrea et al., 2015; Qin et al., 2020), predominantly by forming intra-strand crosslinks with DNA and inhibiting DNA synthesis. Although DNA damage has been suggested as the main mechanism affecting the proliferation of neoplastic cells, more recent data indicate that OxPt may kill cells by inducing ribosome biogenesis stress (Bruno et al., 2017) or by influencing the mitochondrial respiratory chain and energy metabolism (Qin et al., 2020). Regardless of the mechanism of action, OxPt therapy also frequently results in numerous systemic side effects, including those affecting the central and peripheral nervous systems, which force physicians to reduce the dose of medication or discontinue treatment (Branca et al., 2021). Therefore, in recent years many efforts have been made to ameliorate these adverse drug effects to improve the safety profile of OxPt.

The present study demonstrates that an IVLP platform can be used to deliver high doses of OxPt to the lung during surgical resection safely and without inducing systemic toxicity. Furthermore, this work presents precise, minimally-invasive sampling tools for evaluating the pharmacokinetic profile of OxPt within the studied setting, which provides sophisticated information regarding the distribution of the intact drug and its concentrations around the target area—both of which being critical to the efficacy of OxPt-based treatments. Additionally, a comprehensive mapping of cellular disruptions following the administration of chemotherapy was conducted, with the results identifying a number of early predictive biomarkers for cytotoxic chemotherapy-induced lung injury.

A 72-h IVLP porcine survival model was used in this study. Details related to the setting, experimental design (i.e., an accelerated titration dose-escalation study), and an assessment of the subacute toxicities of OxPt within the tested dosage range (5–80 mg/L) have been presented in our recent work (Ramadan et al., 2021). In the current study, three doses of OxPt were explored in detail, with a maximal-tolerated dose of 40 mg/L being administered several times to confirm its safety. Cases at 40 mg/L showed only mild and subclinical lung injury, as manifested by minor histologic and gross changes, as well as limited consolidation on computed tomography, without compromising gas exchange. The novel chemical biopsy approach proposed in this work, which couples SPME and sensitive/accurate analytical instrumentation, was also applied for the pharmacokinetic profiling of OxPt in a novel IVLP-based setup, as well as the comprehensive profiling of disturbances in multiple metabolic pathways.

Knowledge about the cellular pharmacokinetics and/or subcellular distribution of platinum-based anticancer drugs is critical to understanding their pharmacology and toxicity. Several studies on the pharmacokinetics of platinum analogues (cisplatin, carboplatin, and oxaliplatin) have attempted to quantify intact concentrations, or the total Pt, of these drugs. However, these studies have exclusively focused on the metabolic routes taken by these drugs after intravenous (I.V.) administration (Graham et al., 2000; Qin et al., 2020). The present study documents the first comprehensive approach for profiling OxPt and its transient intermediates after administration *via* IVLP and screening the metabolic pathways that are affected as a result. The known pharmacokinetic profile of OxPt during intravenous administration is as follows: within the first hour, the vast majority of the drug rapidly binds to plasma proteins including albumin and gamma-globulins; such binding was found to be moderate and time-dependent, with 85–88% of the total Pt becoming bound within 5 h (Graham et al., 2000). Furthermore, Pt has been shown to irreversibly bind to and accumulate in RBCs. Even though Pt binds to blood cells, it is not considered clinically significant because it represents a minor compartment for drug distribution in patients. For instance, previous studies have demonstrated that approximately 15% of the administered Pt will still be present in the blood at the end of a 2 h infusion. Pt is rapidly cleared from systemic circulation by cellular uptake/covalent binding to tissues and renal elimination. In the context of IVLP, the pharmacokinetics of OxPt is affected by the composition of perfusion fluid, residual blood in the perfusion circuit, and the absence of urinary excretion. Our findings showed significant binding between OxPt and the albumin in the Steen Solution, and a tendency for OxPt to accumulate in RBCs; however, the unbound (free) fraction of the drug seemed to be greater than after I.V. injection. Furthermore, IVLP administration results in OxPt exposure to that is several times higher than after I.V. injection (for detailed explanation, see Ramadan et al., 2021), thus improving the effectiveness of the applied drug/strategy. IVLP administration also minimizes/eliminates some of the adverse effects associated with OxPt frequently observed with I.V. injection, such as haematological toxicity or neuropathy (Di Francesco et al., 2002; Branca et al., 2021). At this point, it is worth noting that there is a clear relationship between the degree of OxPt accumulation in RBCs and the tolerability of therapy (Peng et al., 2005). Indeed, prior analysis of erythrocytes in cancer patients has provided direct evidence that patient prognosis is inverse to the fraction of haemoglobin (Hb) that binds to OxPt; that is, the more haemoglobin that binds to OxPt, the worse the patient’s prognosis. Thus, Hb-OxPt adducts in RBCs can serve as a clinical indicator for toxic response and treatment efficacy.

Undoubtedly, the most intriguing aspect of this study is the proposed sampling approach’s ability to simultaneously monitor spatial and temporal variations in the metabolic patterns of OxPt and several proximate cytotoxic species (monochloro-, dichloro-, and diaquo-DACH platin), along with other noncytotoxic products. Measurements of OxPt levels in tissue captured with Bio-SPME probes showed a rather heterogeneous distribution of the drug (over the course of IVLP) in the two sampled sections of the lung. In particular, higher concentrations of OxPt were observed in the upper lobe, indicating a dose-dependent relationship. The maximal cellular absorption of the drug occurred at the second hour of IVLP (in most cases), followed by a rapid decline to near zero at reperfusion. However, the lower levels of OxPt observed in the lingula were inconsistent with the above-noted trend regarding increased dosages, which suggests that the lingula may not provide representative sampling. This finding is consistent with those of other studies evaluating the reliability and validity of donor tissue biopsies prior to lung transplantation. For instance, Chao et al. (2021) proved that a donor lung biopsy collected from an area other than the lingula can be considered representative of the overall condition of the lung, unless there is obvious localized injury possessing a unique inflammatory-related profile (Chao et al., 2021). Thus, compared to biopsies taken from other sites in donor lungs, the lingula may not provide representative results for diagnostic investigations presenting different physiological pattern.

Furthermore, *in vivo* SPME sampling was also able to provide a representative snapshot of the dynamic changes to the metabolome caused by the IVLP–OxPt treatment. The main advantages offered by SPME, particularly in tissue analysis, include: the ability to tune the geometry of the miniaturized devices to target specific sampling sites; its low invasiveness compared to standard tissue sampling approaches that require biopsy collection; and the non-destructive nature of the extraction procedure. SPME’s miniaturized format, high selectivity for small molecules, and the variety of available biocompatible extraction phases, have positioned it as a convenient and viable strategy for the analysis of a broad range of compounds in diverse matrixes, particularly within the context of global analyte profiling. Indeed, this analytical strategy enabled the identification of multiple pathways that were altered due to the injection of higher doses of OxPt into the perfusion circuit. Specifically, the results indicated the presence of alterations to the metabolism of amino acids, free fatty acids, purine, and pyrimidine over the course of the lung chemo-perfusion, pointing to compromised energy substrate utilization and, presumably, energy deprivation (Pavlova and Thompson, 2016). Amplified oxidative stress manifested by elevated levels of ubiquinone-1 and l-phenylalanine was also recognized in the studied setting. Several previous studies have clearly demonstrated that phenylalanine upregulation in peripheral blood is often the consequence of chronic immune activation, inflammation, and oxidative stress in cancer patients (Lieu et al., 2020). On the other hand, elevated levels of ubiquinone-1 or ubiquinol-6 (the components of mitochondrial respiratory chain) have been characterized as compensatory mechanisms against excessive ROS generation in various pathological conditions (Orr et al., 2013). Furthermore, the upregulation of levels of several bioactive lipid mediators derived from arachidonic acid (pro-inflammatory prostaglandins) or generated from docosahexaenoic and eicosapentaenoic acids (pro-resolving mediators) over the course of IVLP strongly suggests acceleration of the inflammatory response (Serhan, 2014; Sansbury and Spite, 2016; Serhan and Levy, 2018). Our findings demonstrate the dysregulation of a steroidogenic system in a perfused lung, but further research is required to define its overall physiological relevance. Finally, profound alterations in lipid metabolism and/or signalling were also observed. Perturbations in lipid metabolism and, consequently, lipid composition have important therapeutic implications, as they may affect the survival, membrane dynamics, and therapy response of tumour cells (Munir et al., 2019). Changes in the levels of many of the observed lipids may have been due to exposure to metabolically challenging conditions, which is significant, as such adaptation can help cells thrive in harsh microenvironments.

Nonetheless, our study has several important limitations. The main limitation is a relatively small sample size that is directly related with an accelerated titration design. Furthermore, this study’s main goal was to demonstrate the proposed chemical biopsy tool’s usefulness for the non-invasive comprehensive profiling of the metabolic route of anti-cancer drugs and for screening the evolving landscape of the chemotherapy-altered metabolome. Additionally, while the identification of biomarker profiles predictive of lung injury was beyond the scope of this paper, studies are currently in progress that use the proposed analytical approach and larger samples to make clinically relevant observations and identify biomarkers that could aid in the detection of toxicity or inform on therapeutic outcomes. Finally, we explored the cellular distribution of OxPt and its levels in normal tissue and cells, but we did not explore this characteristic in the target cancer cells. Despite these limitations, we ultimately achieved the purpose of this study, which was to provide a rapid estimate of dose-limiting toxicity that can be used as a guide before initiating a safety clinical trial involving metastatic cancer patients.

In conclusion, this paper presented a state-of-the-art analytical pipeline for the comprehensive metabolic profiling of *in vivo* perfused lungs. The proposed method was applied to study the pharmacokinetic behaviour of OxPt in a detailed animal model, including its distribution, effective concentrations, and metabolic route of biotransformation/elimination. In addition to enabling the quantification of the intact drug in diverse biological compartments (lung tissue, perfusate, plasma), Bio-SPME probes enabled us to capture a composite snapshot of the intracellular metabolome. Furthermore, when directly coupled to highly sensitive mass detectors, SPME microprobes can be an invaluable tool for use in rapid diagnostics or tailoring treatments to individual patients. The findings presented herein demonstrate that it is possible to achieve spatially and temporally resolved biochemical/molecular characterization of a living mammalian lung subjected to medical or scientific experimentation or treatment in a fast and minimally invasive manner, thus not disturbing other medical/surgical procedures. Furthermore, SPME technology makes it possible to tailor the analytical protocol to the metabolites or lipids of interest, as it allows researchers to employ more selective extractive phases and desorption parameters. Adding to the above, owing to its miniaturized format, flexibility of design and its easy adaptation to proven analytical approaches, SPME has been positioned as a convenient and viable strategy for *in vivo* analysis with possible future applications directed at providing new insights into the processes characterizing complex biological systems. The sampling devices which potentially can be conveniently coupled to a variety of instrumentations (for rapid determinations) might be customized into a personalized diagnostic tool to support surgeons’ decision-making processes. Along with the evolution of analytical technologies, including portable reading devices using ion mobility devices and optical spectroscopic techniques which might be directly hyphenated to SPME microprobes this technology will continue to advance to address very specific needs of biomedical field. Altogether, that research can be an important reference for researchers conducting pharmacokinetic studies of other anticancer drugs using an IVLP-based route of administration.

## Publisher’s note

All claims expressed in this article are solely those of the authors and do not necessarily represent those of their affiliated organizations, or those of the publisher, the editors and the reviewers. Any product that may be evaluated in this article, or claim that may be made by its manufacturer, is not guaranteed or endorsed by the publisher.

## Data Availability

Metabolomics data associated with this article are available in the EMBL-EBI MetaboLights database (MetaboLights: MTBLS4982).
