# Supplementary material for: The metabolic fate of oxaliplatin in the biological milieu investigated during in vivo lung perfusion using a unique miniaturized sampling approach based on solid-phase microextraction coupled with liquid chromatography-mass spectrometry
Source: Front Cell Dev Biol. 2022 Aug 25;10:928152. doi: 10.3389/fcell.2022.928152 (PMC9453651; doi:10.3389/fcell.2022.928152)
Supplement: Supplementary file 1 [file DataSheet1.docx]

**SUPPLEMENTARY FILE**

**The Metabolic Fate of Oxaliplatin in the Biological Milieu Investigated During *In Vivo* Lung Perfusion Using a Unique Miniaturized Sampling Approach Based on Solid-Phase Microextraction Coupled with Liquid Chromatography-Mass Spectrometry**

Mariola Olkowicz^1^, Hernando Rosales-Solano^1^, Khaled Ramadan^2^, Aizhou Wang^2^, Marcelo Cypel^2,3^, Janusz Pawliszyn^1†^

^1^ Department of Chemistry, University of Waterloo, Waterloo, ON, Canada;

^2^ Latner Thoracic Surgery Research Laboratories, Toronto General Hospital Research Institute, University Health Network, Toronto, ON, Canada;

^3^ Division of Thoracic Surgery, Department of Surgery, University Health Network, University of Toronto, Toronto Lung Transplant Program, Toronto, ON, Canada

† Corresponding author:

janusz@uwaterloo.ca (J. Pawliszyn), Tel: +1 519-888-4641, Fax: +1 519-888-4348

Dichloro-DACH platinum

Oxaliplatin

Diaquo-DACH platinum

Carboplatin (IS)

**Sup. Fig. S1.** Representative extracted-ion SRM chromatograms of oxaliplatin, its metabolites, and an internal standard (carboplatin) obtained *via* LC-MS/MS analysis of a selected quality control sample.

Dichloro-DACH platinum –

trace amounts

Oxaliplatin

Diaquo-DACH platinum –

not detected

Carboplatin (IS)

**Sup. Fig. S2.** Representative extracted-ion SRM chromatograms of oxaliplatin, its metabolites, and an internal standard (carboplatin) obtained *via* LC-MS/MS analysis of a selected supernatant sample (2^nd^ hour of perfusion).

Dichloro-DACH platinum –

trace amounts

Oxaliplatin

Diaquo-DACH platinum –

not detected

Carboplatin (IS)

**Sup. Fig. S3.** Representative extracted-ion SRM chromatograms of oxaliplatin, its metabolites, and an internal standard (carboplatin) obtained *via* LC-MS/MS analysis of a selected raw perfusate sample (2^nd^ hour of perfusion).

Dichloro-DACH platinum –

trace amounts

Oxaliplatin

Diaquo-DACH platinum –

not detected

Carboplatin (IS)

**Sup. Fig. S4.** Representative extracted-ion SRM chromatograms of oxaliplatin, its metabolites, and an internal standard (carboplatin) obtained *via* LC-MS/MS analysis of a selected lung extract (1^st^ hour of perfusion, upper lobe).

**A Lung/ESI+ B**


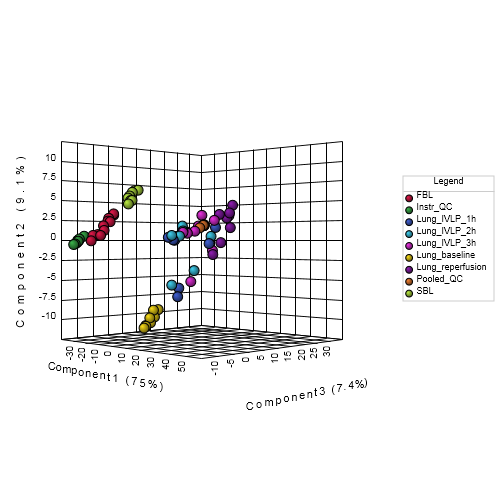

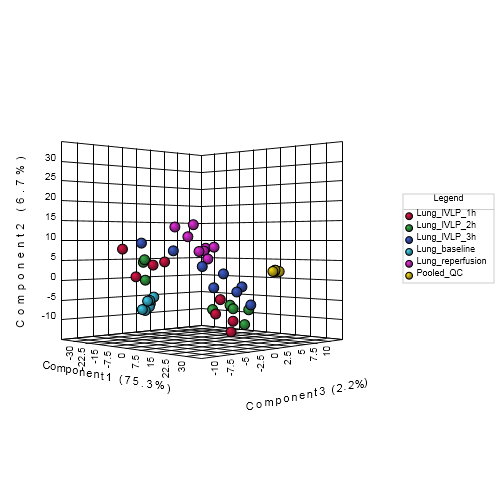


**C Lung/ESI- D**


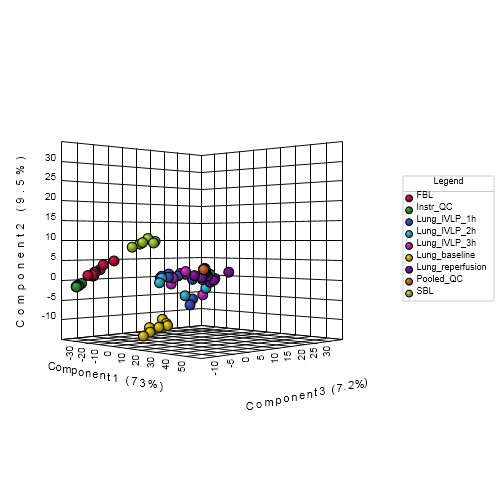

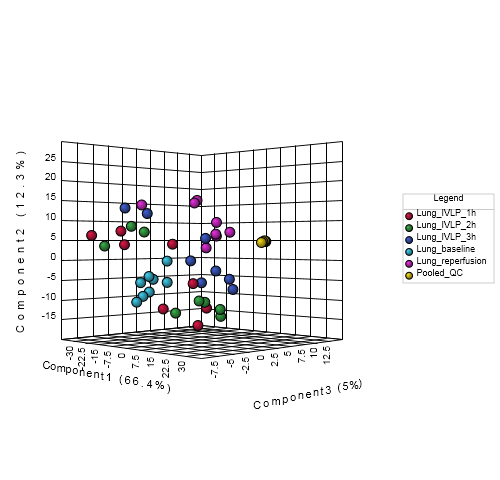


**Sup. Fig. S5.** PLS-DA score plots for all metabolite features detected during **lung sampling** comparing metabolomics profiles for 5 studied conditions (pre-perfusion; at the 1^st^, 2^nd^, and 3^rd^ hour of IVLP; and reperfusion). The results showed that the QC (quality control) samples clustered in the center (pooled QCs) and segregated into a tight cluster (pooled QCs or instrumental QCs) in the relevant score plots in either positive or negative ionization mode. **Instr_QC**: instrumental QC samples. **Pooled_QC**: pooled QC samples. **FBL/SBL**: fiber/solvent blank samples. **Lung_baseline**: samples collected at pre-perfusion. **Lung_IVLP_1h/2h/3h**: samples collected at the 1^st^, 2^nd^, and 3^rd^ hour of IVLP. **Lung_reperfusion**: samples collected 30 min post reperfusion. **ESI+**: positive ionization mode. **ESI-**: negative ionization mode.

**A SN/MM/ESI+ B**


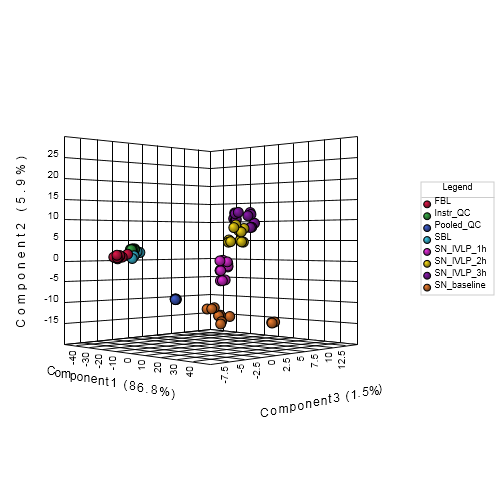

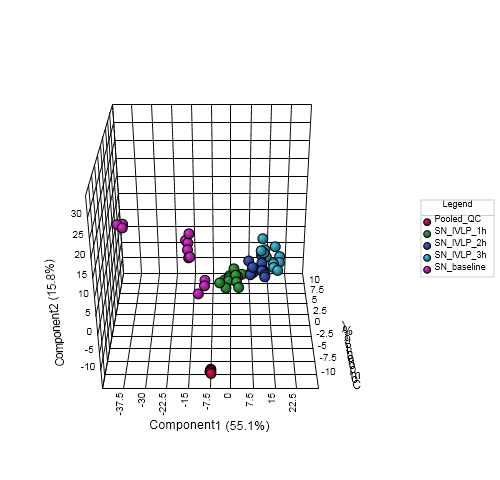


**C SN/MM/ESI- D**


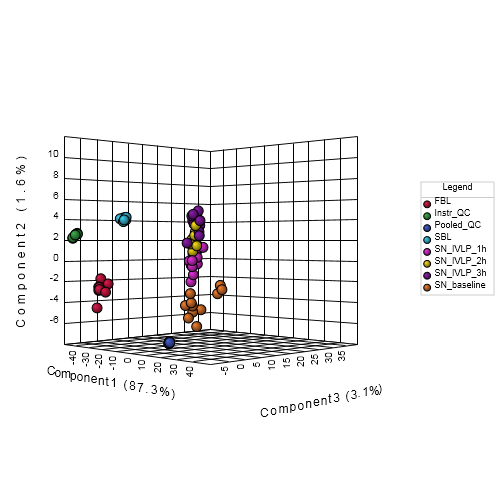

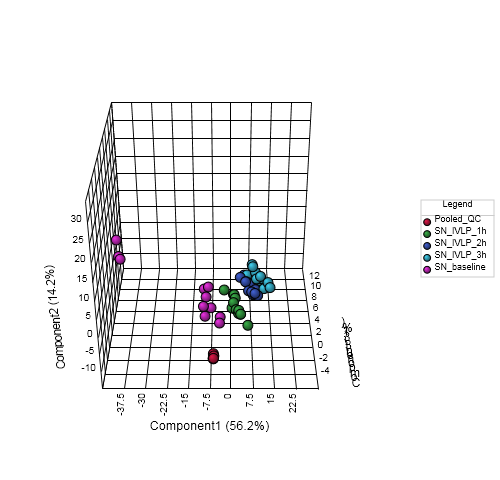


**Sup. Fig. S6.** PLS-DA score plots for all metabolite features detected during **supernatant sampling** comparing metabolomics profiles for 4 studied conditions (pre-perfusion (before drug administration) and at the 1^st^, 2^nd^, and 3^rd^ hour of IVLP). The tight clustering of quality control samples—both pooled QCs (in the plot center) and instrumental QCs—indicates excellent instrument reproducibility during the entire run. The presented plots were created for the features detected in positive (**A**, **B**) and negative (**C**, **D**) ion modes when the mixed-mode (MM) coating was used for extraction and PFP chromatographic mode was used for analyte separation. **SN**: supernatant samples. **Instr_QC**: instrumental QC samples. **Pooled_QC**: pooled QC samples. **FBL/SBL**: fiber/solvent blank samples. **SN_baseline**: samples collected before OxPt administration. **SN_IVLP_1h/2h/3h**: samples collected at the 1^st^, 2^nd^, and 3^rd^ hour of IVLP. **ESI+**: positive ionization mode. **ESI-**: negative ionization mode.

**A SN/C18/ESI+ B**


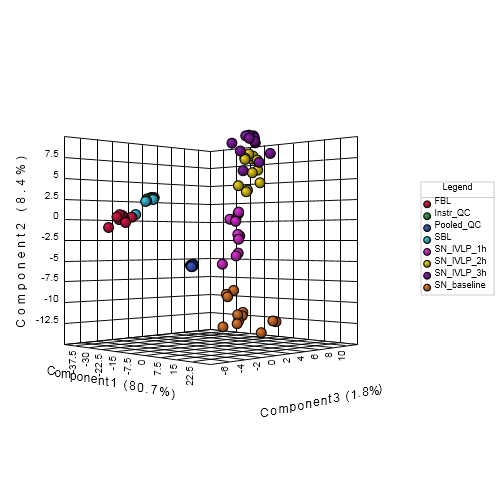

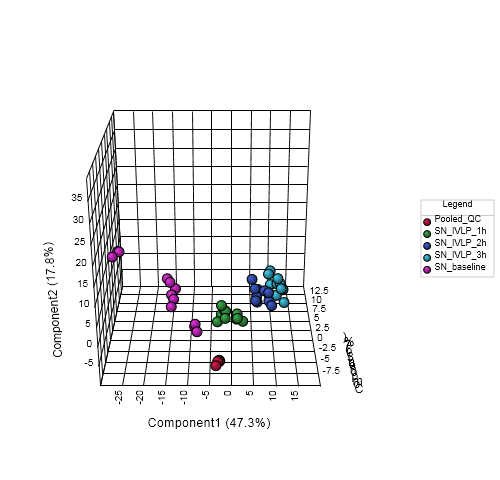


**C SN/C18/ESI- D**


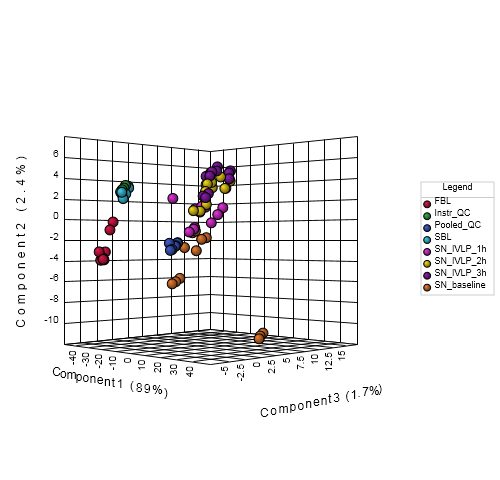

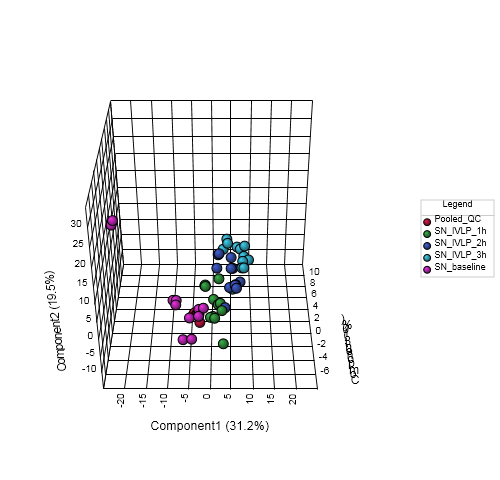


**Sup. Fig. S7.** PLS-DA score plots for all features detected during **supernatant sampling** comparing lipidomics profiles for 4 studied conditions (pre-perfusion (before drug administration), and at the 1^st^, 2^nd^, and 3^rd^ hour of IVLP). The clustering of QC sample data points is indicative of good reproducibility. The presented plots were created for the features detected in positive (**A**, **B**) and negative (**C**, **D**) ion modes when the C18 coating was used for analyte extraction and reversed phase (RP) chromatographic mode was used for analyte separation. **SN**: supernatant samples. **Instr_QC**: instrumental QC samples. **Pooled_QC**: pooled QC samples. **FBL/SBL**: fiber/solvent blank samples. **SN_baseline**: samples collected before OxPt administration. **SN_IVLP_1h/2h/3h**: samples collected at the 1^st^, 2^nd^, and 3^rd^ hour of IVLP. **ESI+**: positive ionization mode. **ESI-**: negative ionization mode.

**A RP/MM/ESI+ B**


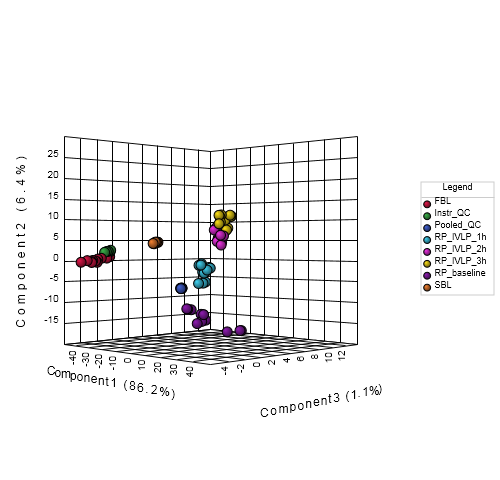

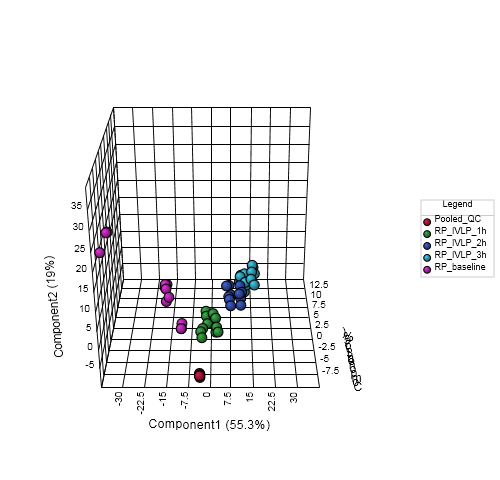


**C D**


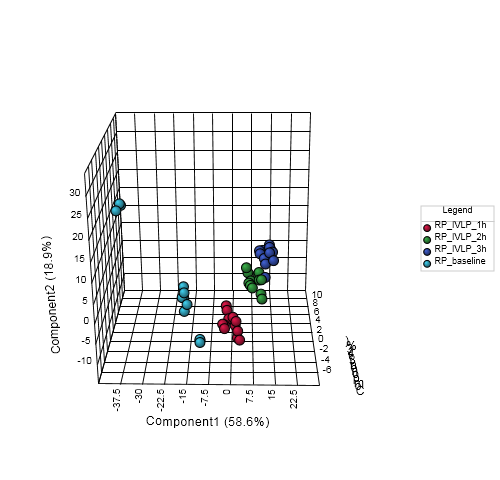

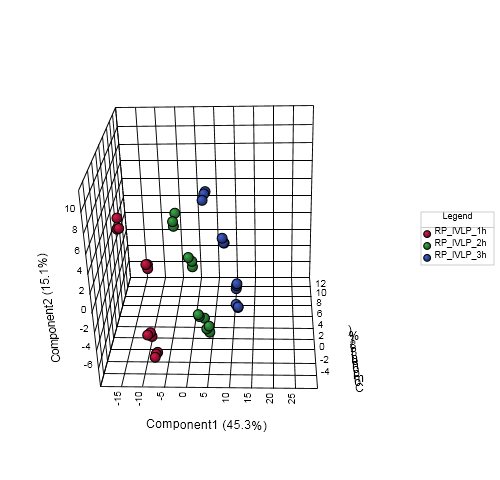


**Sup. Fig. S8.** PLS-DA score plots for all metabolite features detected during **raw perfusate sampling** comparing metabolomics profiles for 4 studied conditions (pre-perfusion (before drug administration), and at the 1^st^, 2^nd^, and 3^rd^ hour of IVLP). As can be seen, the quality control samples cluster tightly together in the plots. The models were created for the features detected in positive ion mode (**ESI+**) when the mixed-mode (MM) coating was used for extraction and PFP chromatographic mode was used for analyte separation. **RP**: raw perfusate samples. **Instr_QC**: instrumental QC samples. **Pooled_QC**: pooled QC samples. **FBL/SBL**: fiber/solvent blank samples. **RP_baseline**: samples collected before OxPt administration. **RP_IVLP_1h/2h/3h**: samples collected at the 1^st^, 2^nd^, and 3^rd^ hour of IVLP.

**A RP/MM/ESI- B**


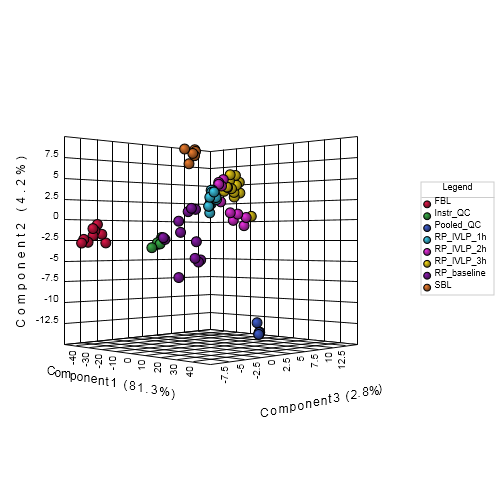

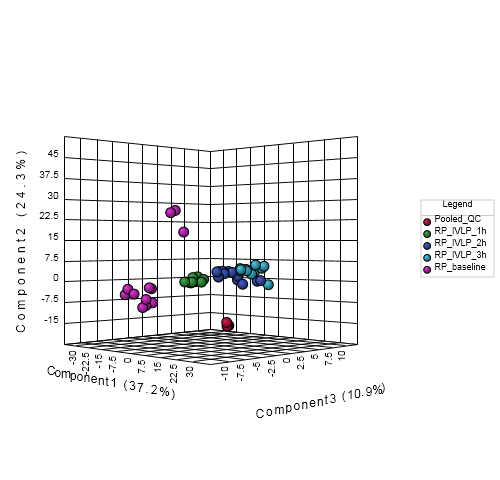


**C D**


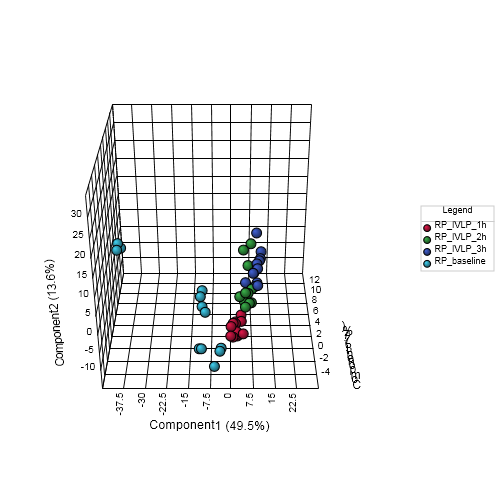

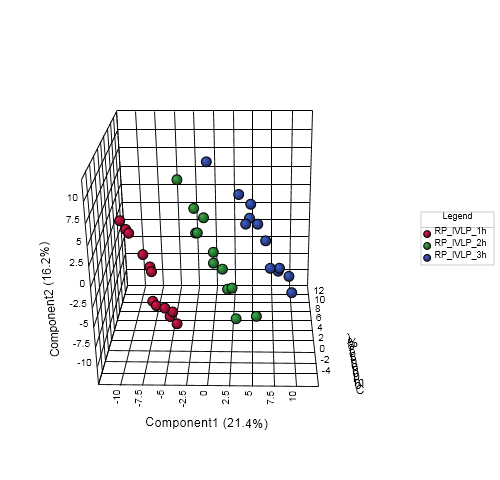


**Sup. Fig. S9.** PLS-DA score plots for all metabolite features detected during **raw perfusate sampling** comparing metabolomics profiles for 4 studied conditions (pre-perfusion (before drug administration), and at the 1^st^, 2^nd^, and 3^rd^ hour of IVLP). As can be seen, the quality control samples are clustered tightly together in the plots. The models were created for the features detected in negative ion mode (**ESI-**) when the mixed-mode (MM) coating was used for extraction and PFP chromatographic mode was used for analyte separation. **RP**: raw perfusate samples. **Instr_QC**: instrumental QC samples. **Pooled_QC**: pooled QC samples. **FBL/SBL**: fiber/solvent blank samples. **RP_baseline**: samples collected before OxPt administration. **RP_IVLP_1h/2h/3h**: samples collected at the 1^st^, 2^nd^, and 3^rd^ hour of IVLP.

**A RP/C18/ESI+ B**


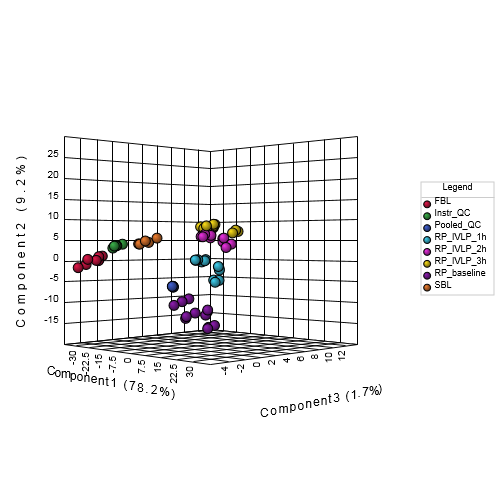

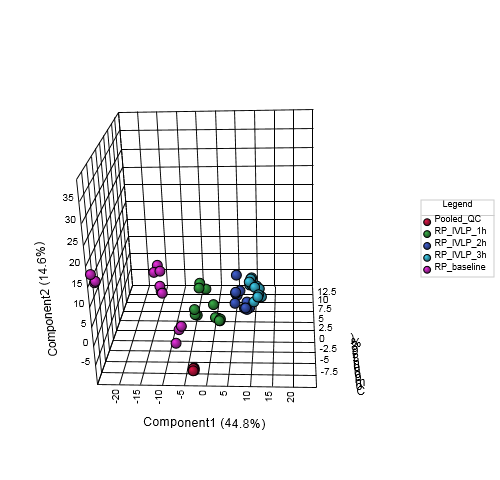


**C D**


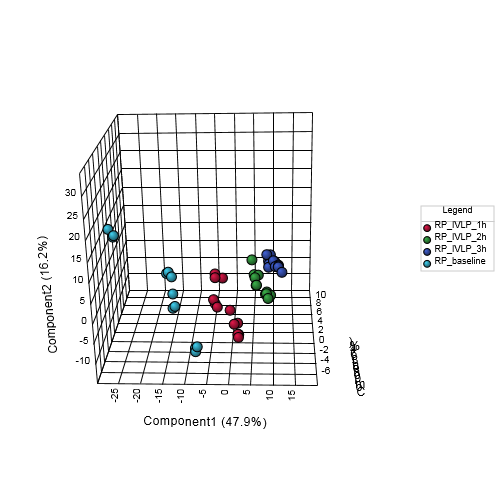

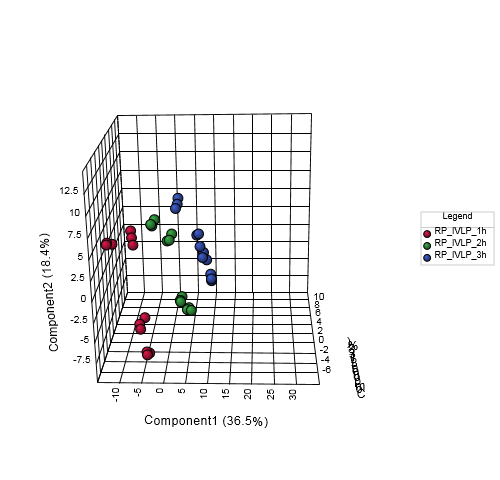


**Sup. Fig. S10.** PLS-DA score plots for all features detected during **raw perfusate sampling** comparing lipidomics profiles for 4 studied conditions (pre-perfusion (before drug administration), and at the 1^st^, 2^nd^, and 3^rd^ hour of IVLP). As can be seen, the quality control samples are clustered tightly together in the plots. The models were created for the features detected in positive ion mode (**ESI+**) when the C18 coating was used for extraction and RP chromatographic mode was used for analyte separation. **RP**: raw perfusate samples. **Instr_QC**: instrumental QC samples. **Pooled_QC**: pooled QC samples. **FBL/SBL**: fiber/solvent blank samples. **RP_baseline**: samples collected before OxPt administration. **RP_IVLP_1h/2h/3h**: samples collected at the 1^st^, 2^nd^, and 3^rd^ hour of IVLP.

**A RP/C18/ESI- B**


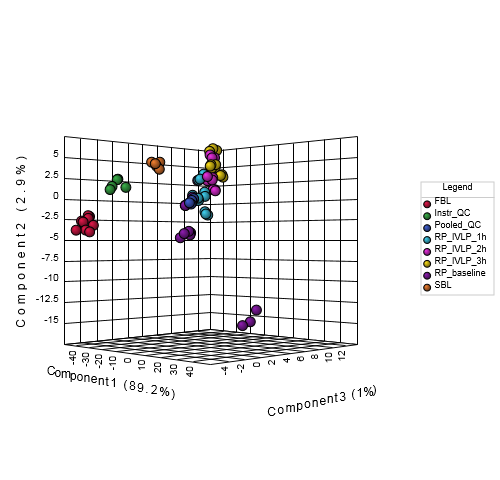

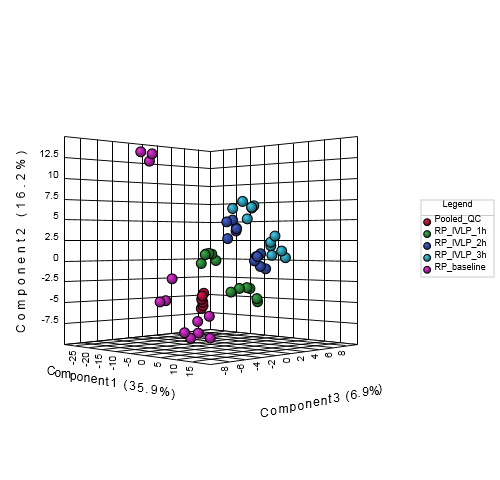


**C D**


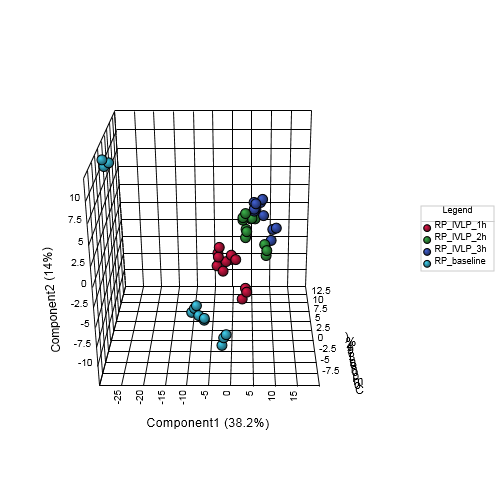

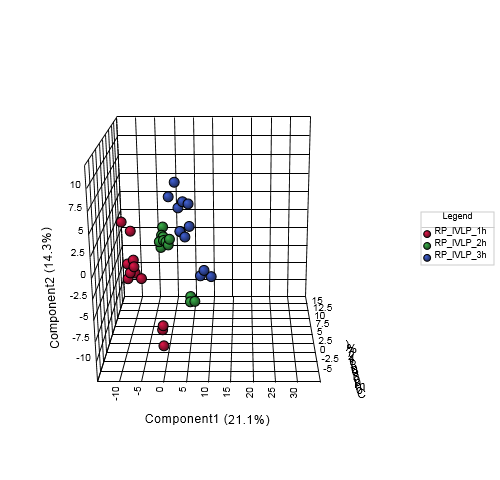


**Sup. Fig. S11.** PLS-DA score plots for all features detected during **raw perfusate sampling** comparing lipidomics profiles for 4 studied conditions (pre-perfusion (before drug administration), and at the 1^st^, 2^nd^, and 3^rd^ hour of IVLP). As can be seen, the quality control samples are clustered tightly together in the plots. The models were created for the features detected in negative ion mode (**ESI-**) when the C18 coating was used for extraction and RP chromatographic mode was used for analyte separation. **RP**: raw perfusate samples. **Instr_QC**: instrumental QC samples. **Pooled_QC**: pooled QC samples. **FBL/SBL**: fiber/solvent blank samples. **RP_baseline**: samples collected before OxPt administration. **RP_IVLP_1h/2h/3h**: samples collected at the 1^st^, 2^nd^, and 3^rd^ hour of IVLP.

**Sup. Table S1.** Top 16 metabolites and lipid species (along with their putative identification) exhibiting significant alterations in the lung during *in vivo* chemo-perfusion.

| No. | Putative  identification | m/z | Rt (min) | Adduct | Δ ppm* | Metabolic  pathway |
| --- | --- | --- | --- | --- | --- | --- |
| 1  2  3  4  5  6  7  8  9  10  11  12  13  14  15  16 | N-acetyl-L-tryptophan  Indolelactic Acid  6-hydroxyindolelactate  Ubiquinone-1  Resolvin D1-D4  Malonylcarnitine  Cyclic phospatidic acid (18:0)  3’/5’-CMP  3’/5’-CMP  L-Phenylalanine  Adenosine  11-dehydrocorticosterone  18-OH-corticosterone  Aldosterone  Phosphatidylinositol (12:0)  Creatine  5′-Deoxy-5′-(methylthio)adenosine | 247.1077  269.0897  263.1026  268.1544  377.2324  289.1386  459.2283  324.0591  324.0591  166.0864  268.1041  343.1914  343.1915  419.2078  515.2260  132.0768  298.0976 | 13.67  13.67  13.68  16.61  14.81  15.73  17.20  12.07  12.34  11.10  9.13  17.15  15.77  15.77  15.77  2.12  18.04 | [M+H] ^+^  [M+ACN+Na] ^+^  [M+ACN+H] ^+^  [M+NH_4_] ^+^  [M+H] ^+^  [M+ACN+H] ^+^  [M+K] ^+^  [M+H] ^+^  [M+H] ^+^  [M+H] ^+^  [M+H] ^+^  [M-H] ^–^  [M-H_2_O-H] ^–^  [M+CH_3_COO] ^–^  [M-H] ^–^  [M+H] ^+^  [M+H] ^+^ | 0  0  0  0  0  2  2  0  0  0  0  0  1  0  0  0  2 | *Trp metabolism*  *Trp metabolism*  *Trp metabolism*  *Oxidative stress*  *Inflammatory response*  *FFA metabolism*  *Tissue injury*  *Purine metabolism*  *Purine metabolism*  *AA metabolism*  *Purine metabolism*  *Aldosterone synthesis*  *Aldosterone synthesis*  *Aldosterone synthesis*  *Cellular signaling*  *ATP generation*  *Purine metabolism* |

*mass accuracy (mass error).

**A**
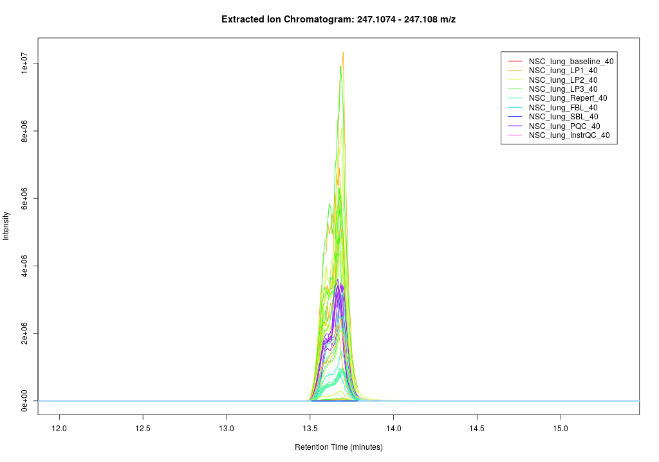
**B**
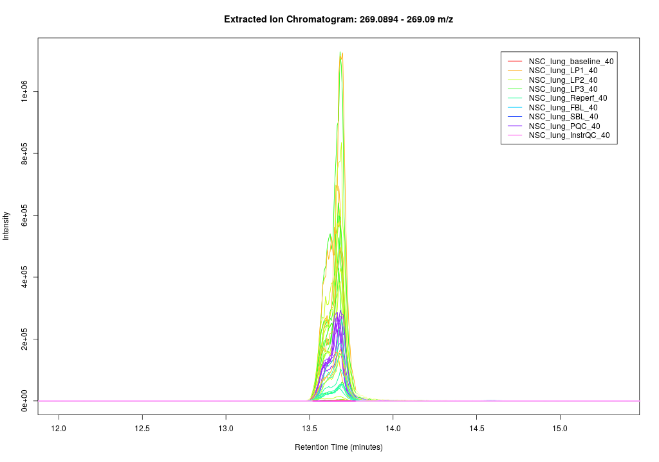


**C**
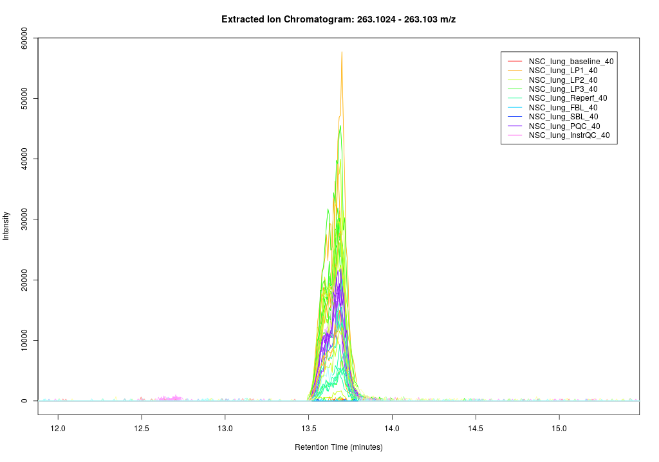
**D**
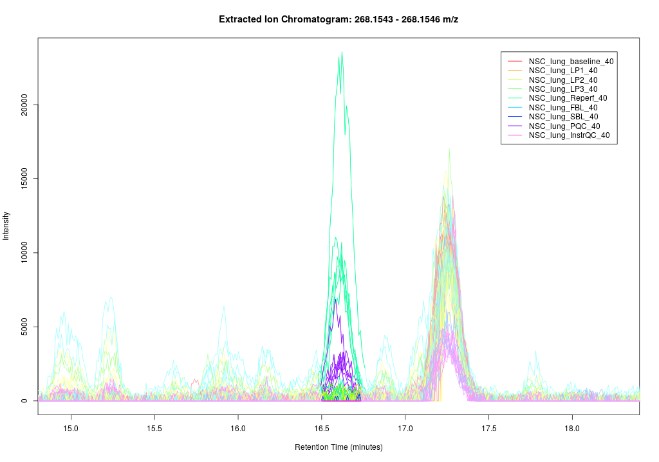


**E**
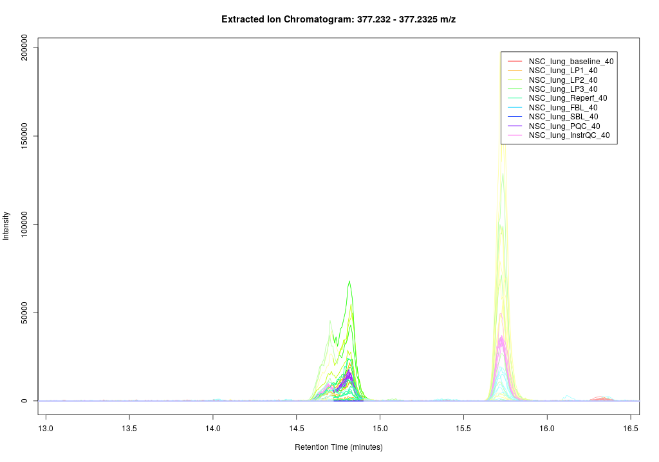
**F**
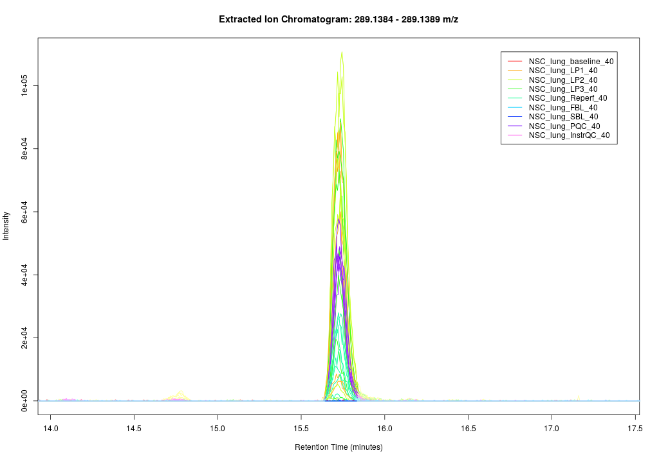


**G**
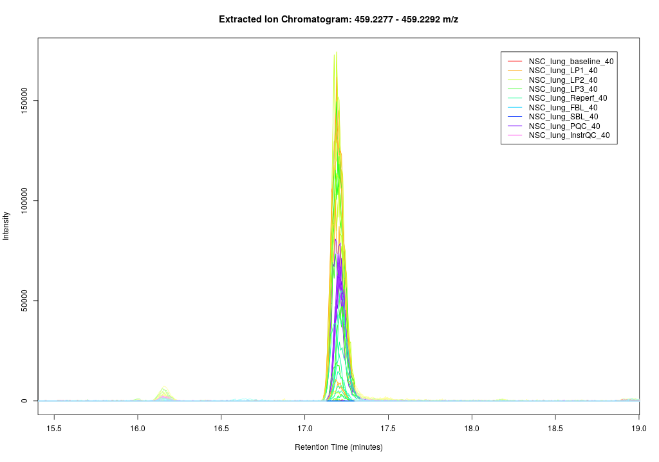
**H**
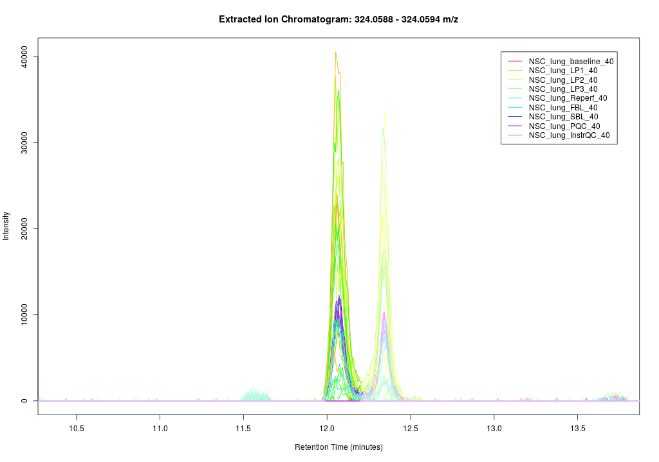


**I**
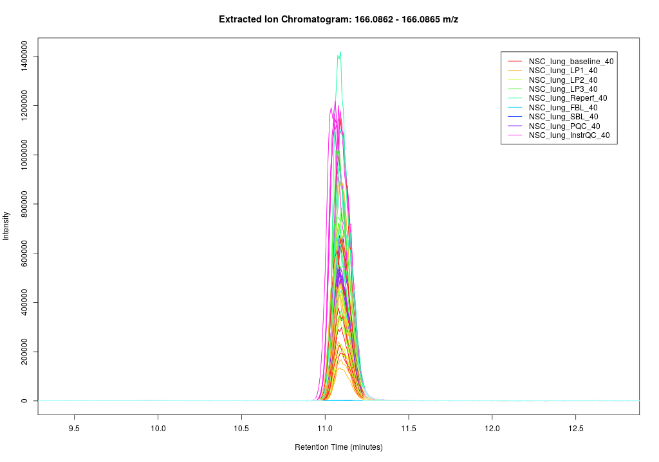
**J**
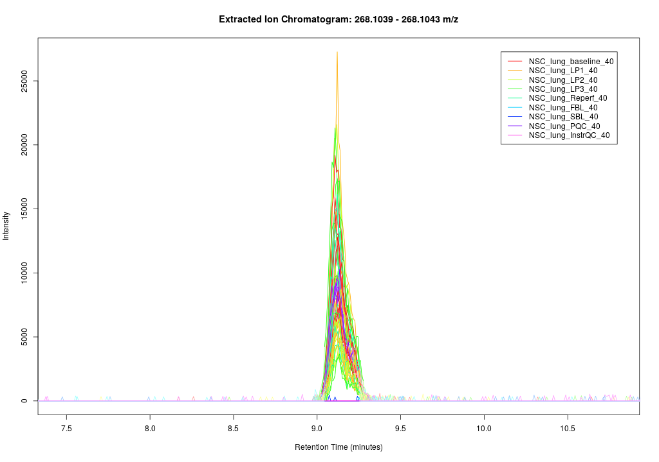


**K**
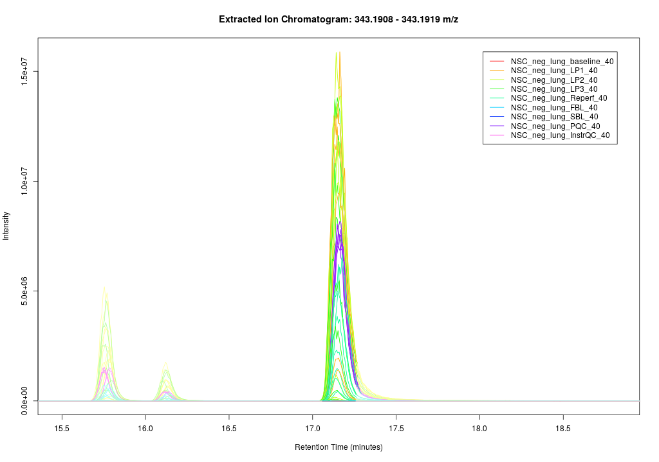
**L**
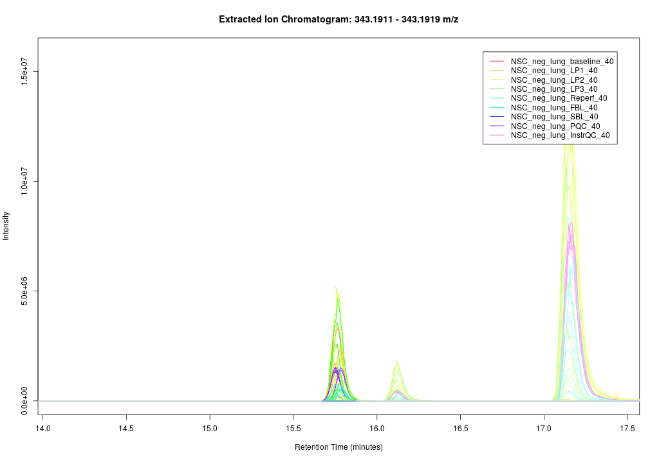


**M**
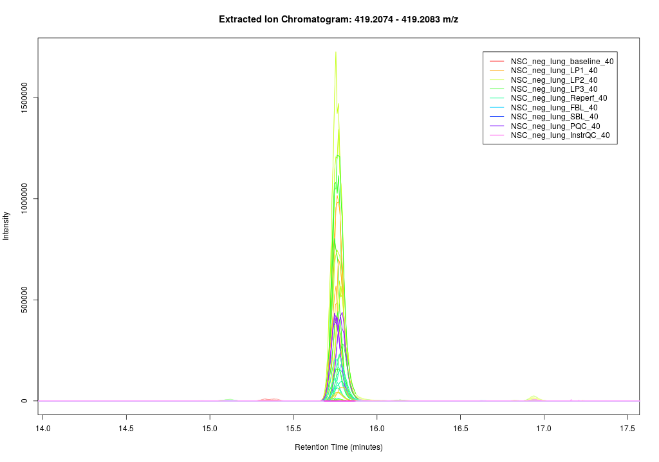
**N**
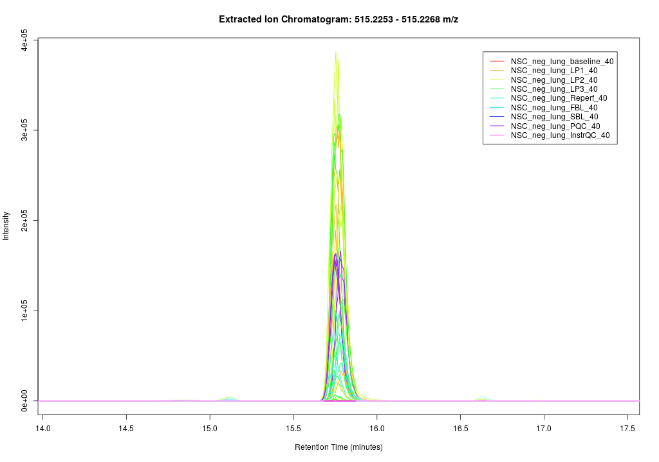


**O**
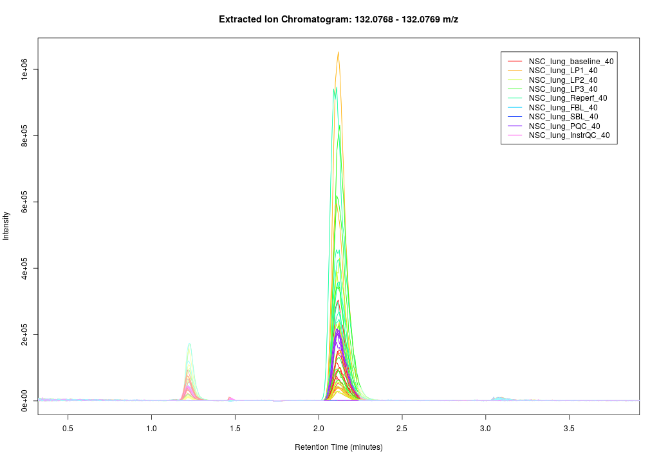
**P**
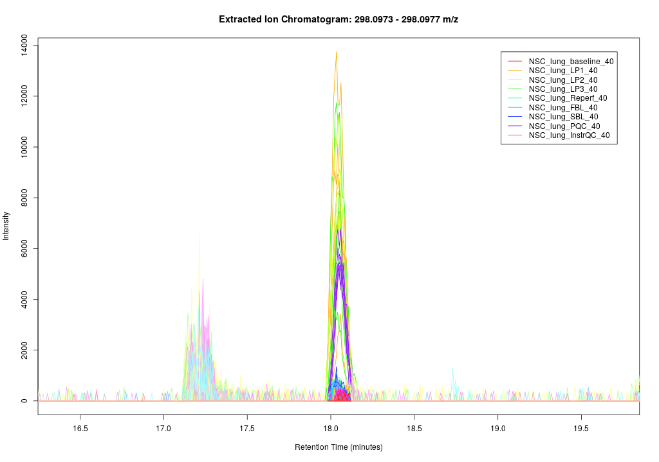


**Sup. Fig. S12.** Extracted ion chromatograms for the metabolites and lipids presented in Sup. Table S1.

**Sup. Table S2.** Top 32 metabolites and lipid species (along with their putative identification) exhibiting significant alterations in the perfusate during *in vivo* chemo-perfusion.

| No. | Putative  identification | m/z | Rt (min) | Adduct | Δ ppm* | Metabolic  pathway |
| --- | --- | --- | --- | --- | --- | --- |
| 1  2  3  4  5  6  7  8  9  10  11  12  13  14  15  16  17  18  19  20  21  22  23  24  25  26  27  28  29  30  31  32 | **MM-SPME probes**  1-Heptanoyl-sn-glycero-3-phosphocholine (LPC (7:0))  Glycochenodeoxycholate-3/7-sulfate  L-Phenylalanine  5′-Deoxy-5′-(methylthio)adenosine  Ceramide phosphate (d27:1)  Lysophosphatidyl-ethanolamine (10:0)  2-Aminoadenosine  5’-Dehydroadenosine  L-Histidine  1-Octadecyl-sn-glycero-3-phosphoinositol-3-phosphate (LPIP (O-18:0)  Hydroxycaprylic acid  Phosphoethanolamine-ceramide (t35:0)  Phosphoethanolamine-ceramide (d31:0)  Phosphatidic acid (35:2)  Ubiquinone-1  Trihydroxystearic acid  Thiamine pyrophosphate  **C18-SPME probes**  PGP (PGF1alpha/i-12:0)  PGP (22:5-O(16,17)/i-13:0)  PA (18:1-2OH/22:1)  L-histidinol-phosphate  Hydroxypropionylcarnitine  N-stearoyl arginine  Ubiquinone-1  Leukotriene B5/  Prostaglandin J2/  12-Keto-leukotriene B4  Octanoylcarnitine  PE (LTE4/15:0)  PG (44:7)  PG (42:5)  Deoxycytidine  Stearoylcarnitine  Docosahexaenoic acid  Docosapentaenoic acid (22n-3)/(22n-6) | 433.2068  494.2595  166.0864  298.0975  537.3990  392.1737  283.1153  283.1153  156.0769  665.3029  159.1020  751.5582  679.5005  667.4698  249.1132  391.2703  893.0702  435.2133  887.3883  872.6120  239.0891  234.1337  468.3895  233.1174  335.2217  288.2170  450.2334  894.6256  894.6256  455.1888  464.3147  327.2331  329.2487  329.2487 | 16.71  16.72  11.12  18.06  17.20  16.96  13.33  13.33  1.97  16.58  14.58  19.22  18.62  20.85  19.31  15.27  14.26  0.81  0.66  10.26  0.71  0.74  8.59  9.89  9.89  0.87  0.82  10.22  10.22  0.70  11.05  10.98  11.35  11.63 | [M+ACN+Na] ^+^  [M+H-2H_2_O] ^+^  [M+H] ^+^  [M+H] ^+^  [M+NH_4_] ^+^  [M+Na] ^+^  [M+H] ^+^  [M+NH_4_] ^+^  [M+H] ^+^  [M-H] ^–^  [M-H] ^–^  [M+CH_3_COO] ^–^  [M+CH_3_COO] ^–^  [M-H_2_O-H] ^–^  [M-H] ^–^  [M+CH_3_COO] ^–^  [2M+FA-H] ^–^  [M+H+Na] ^2+^  [M+K] ^+^  [M+iPrOH+  Na+H] ^2+^  [M+NH_4_] ^+^  [M+H] ^+^  [M+ACN+H] ^+^  [M+H-H_2_O] ^+^  [M+H] ^+^  [M+H] ^+^  [M+H+K] ^2+^  [M+NH_4_] ^+^  [M+ACN+H] ^+^  [2M+H] +  [M+K-2H] ^–^  [M-H] ^–^  [M-H] ^–^  [M-H] ^–^ | 1  2  0  2  0  0  1  1  0  0  4  0  0  0  0  0  4  1  1  0  5  0  3  1  0  0  2  4  4  1  0  0  0  0 | *Cellular signaling*  *Bile acid metabolism*  *AA metabolism*  *Purine metabolism*  *Lipid metabolism*  *Lipid metabolism*  *Purine metabolism*  *Purine metabolism*  *AA metabolism*  *Lipid metabolism*  *FFA metabolism*  *Lipid metabolism*  *Lipid metabolism*  *Cellular signaling*  *Oxidative stress*  *Lipid metabolism*  *Peroxisomal metabolism*  *Cellular signaling*  *Cellular signaling*  *Cellular signaling*  *Histidine biosynthesis*  *FFA oxidation*  *AA metabolism*  *Oxidative stress*  *Inflammatory response*  *FFA oxidation*  *Cellular signaling*  *Cellular signaling*  *Cellular signaling*  *Pyrimidine metabolism*  *FFA metabolism*  *Lipid metabolism*  *Lipid metabolism*  *Lipid metabolism* |

**A**
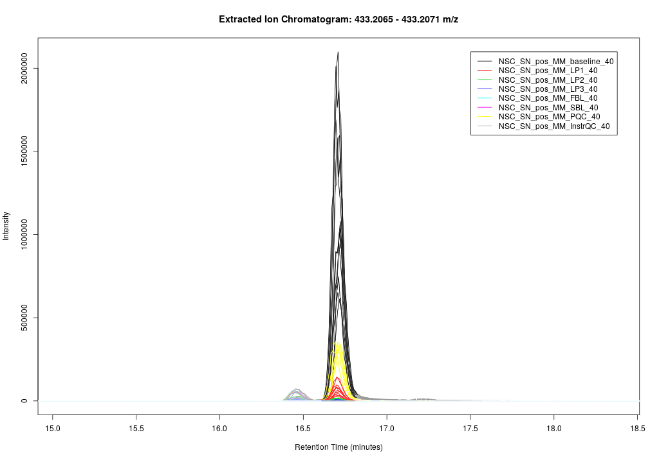
**B**
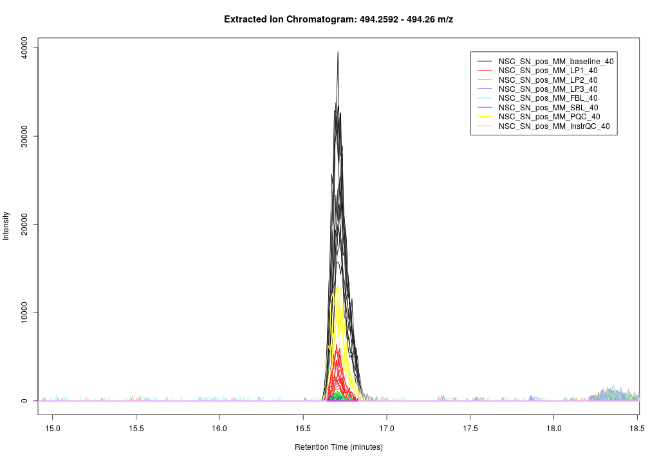


**C**
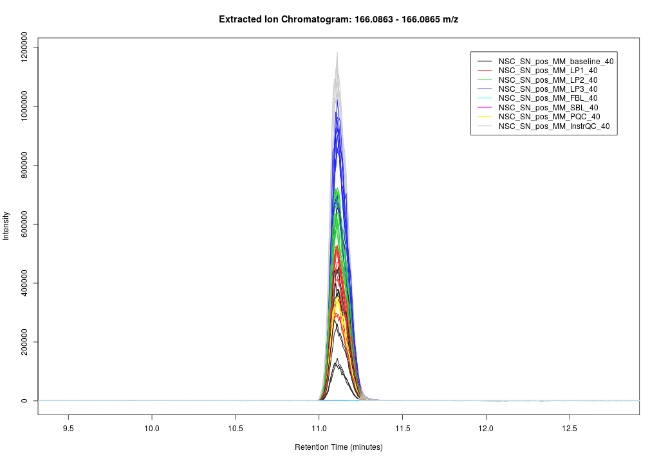
**D**
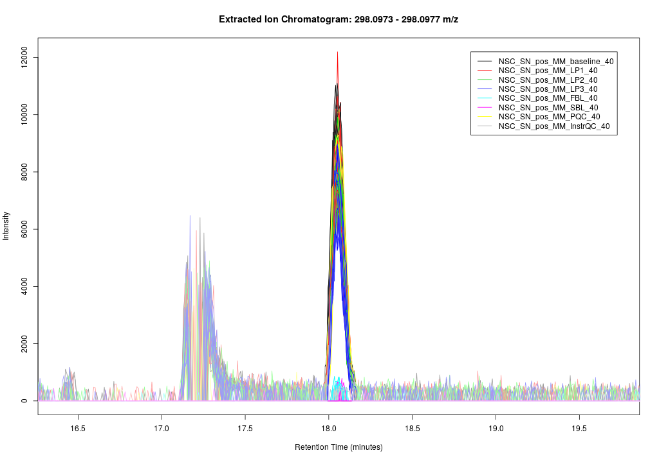


**E**
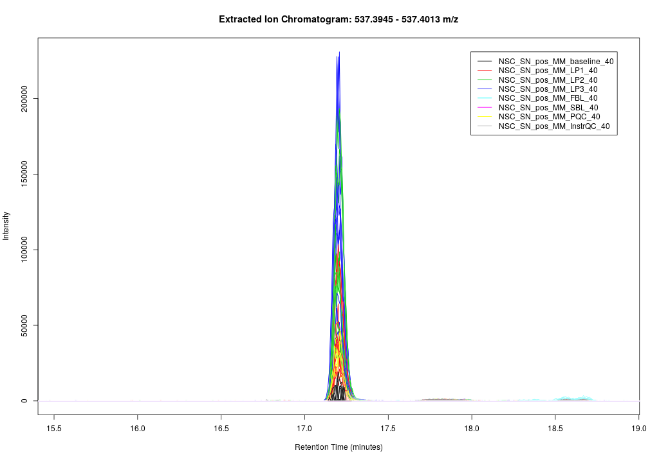
**F**
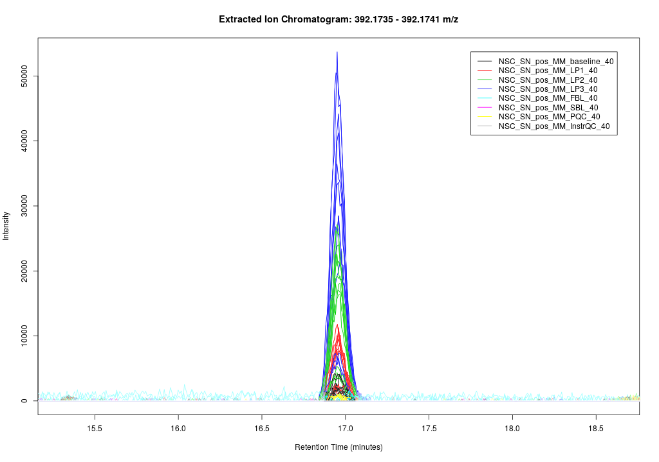


**G**
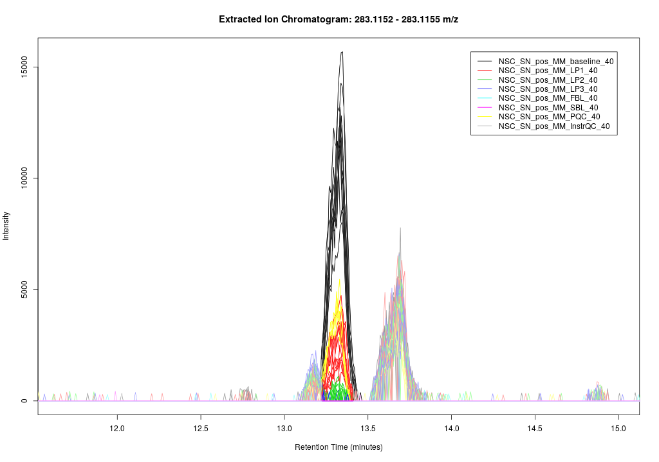
**H**
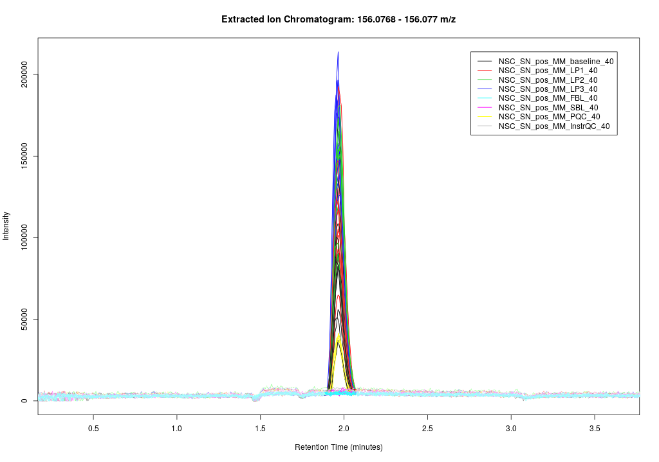


**I**
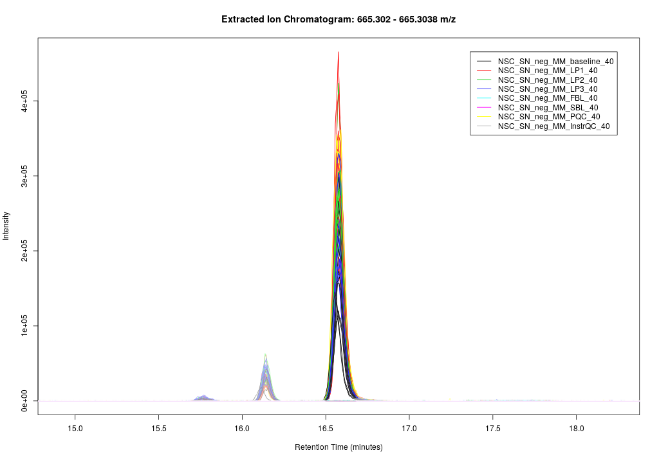
**J**
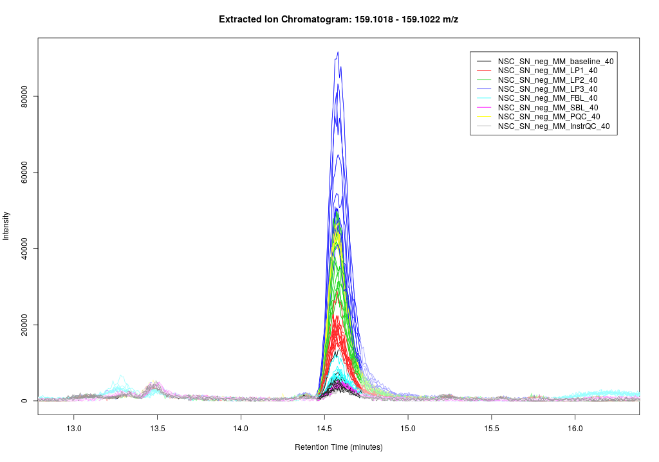


**K**
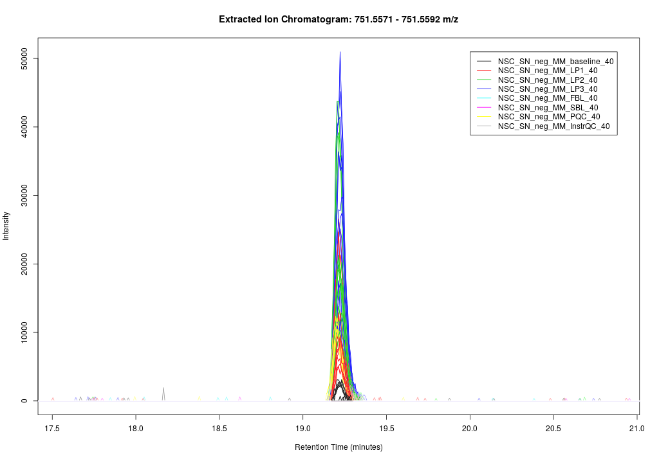
**L**
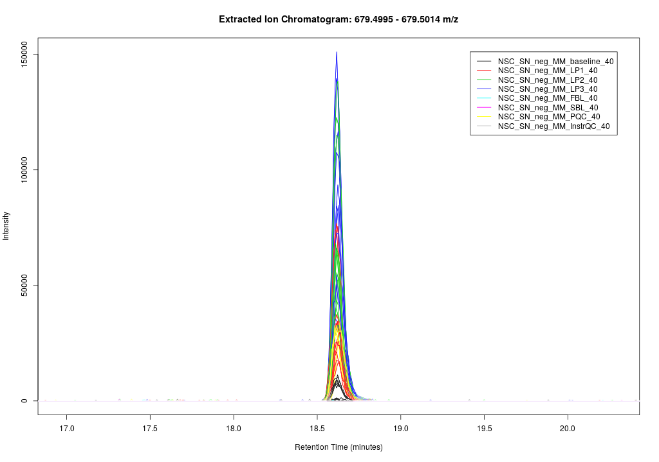


**M**
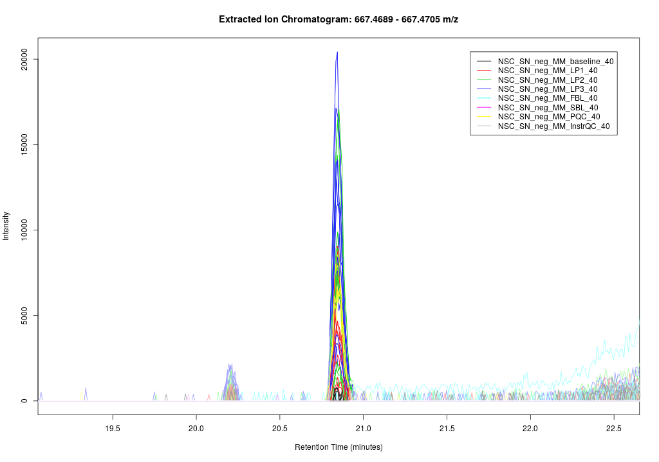
**N**
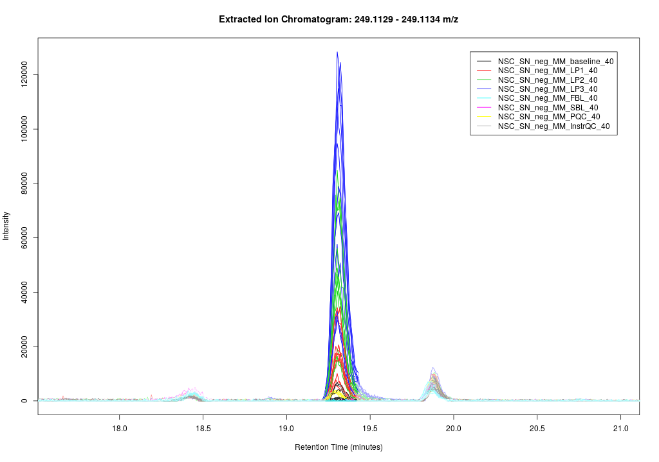


**O**
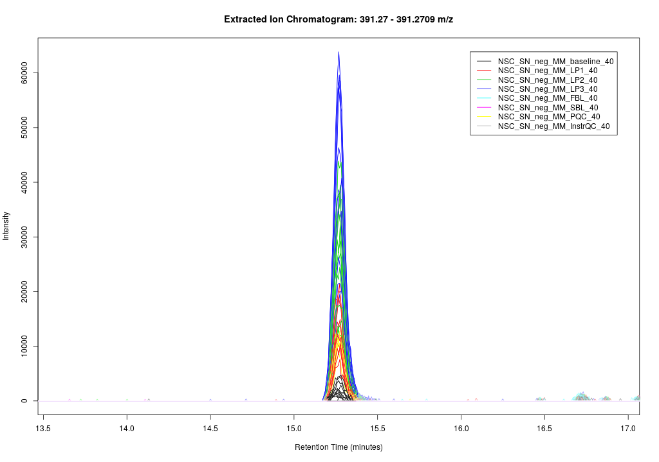
**P**
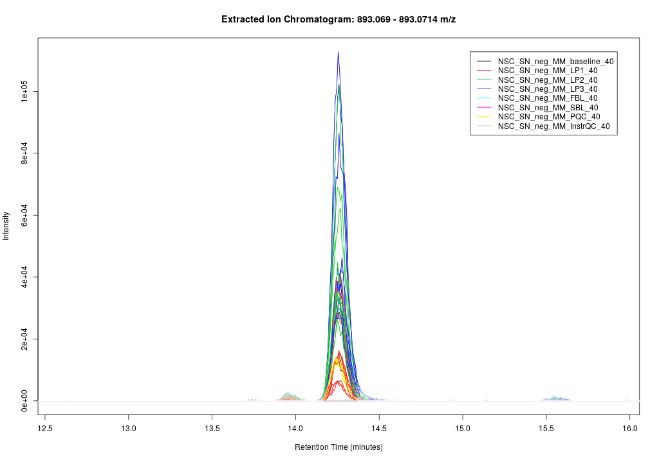


**Sup. Fig. S13.** Extracted ion chromatograms of selected metabolites and lipids presented in Sup. Table S2 extracted with MM probes.

**A**
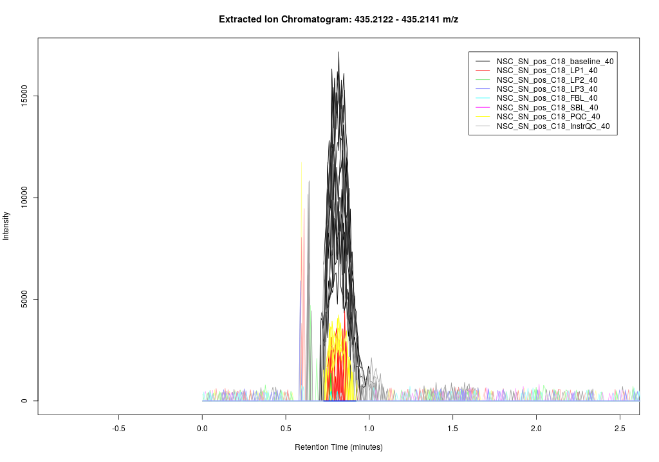
**B**
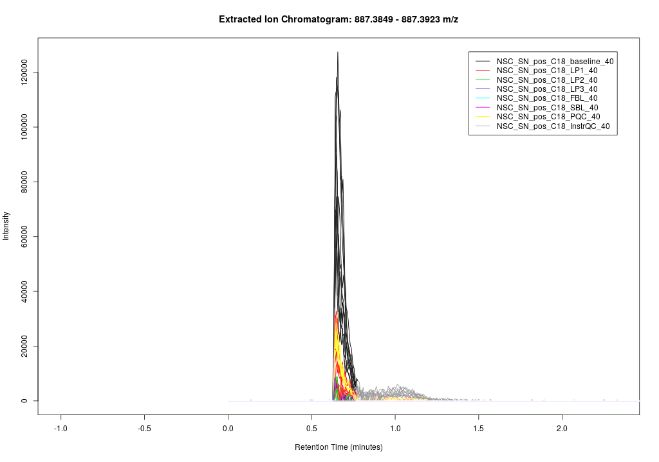


**C**
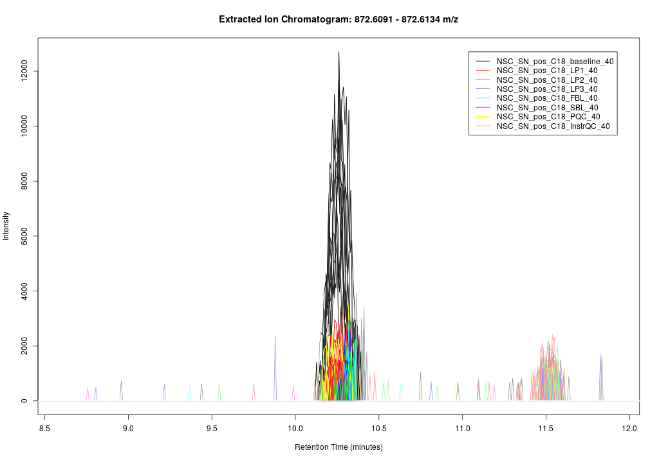
**D**
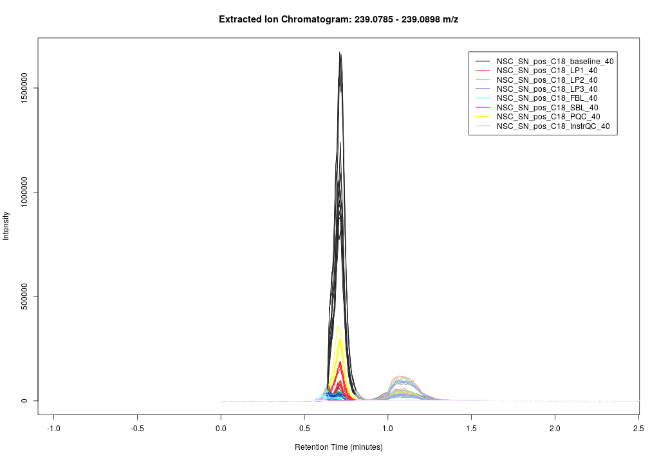


**E**
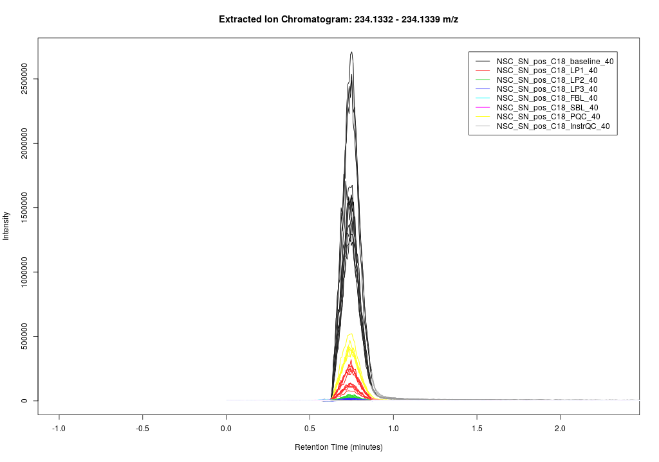
**F**
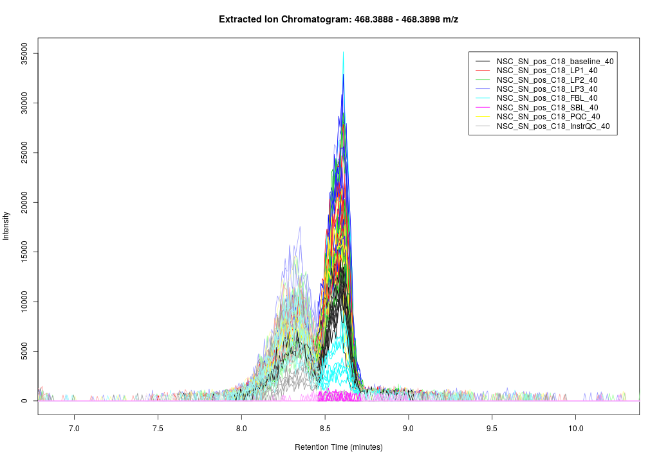


**G**
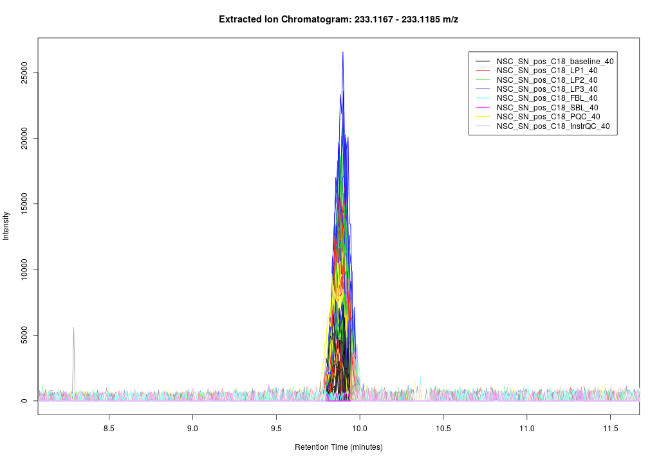
**H**
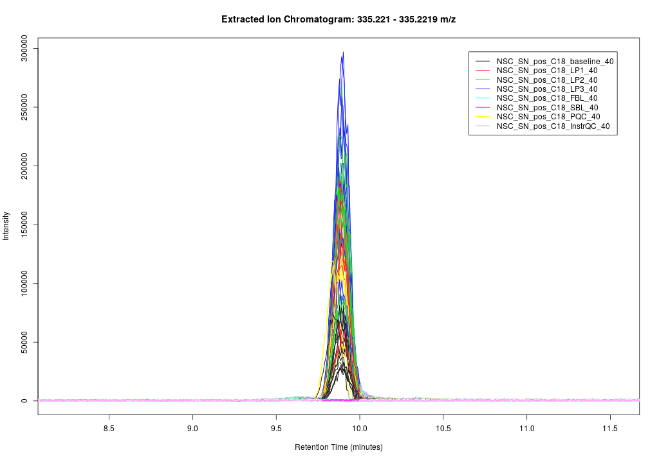


**I**
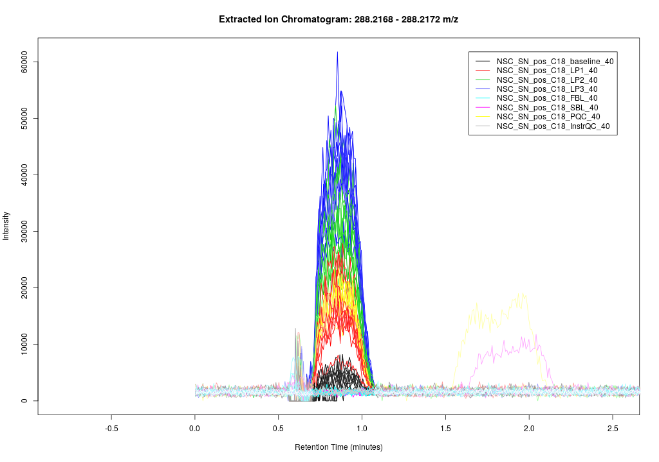
**J**
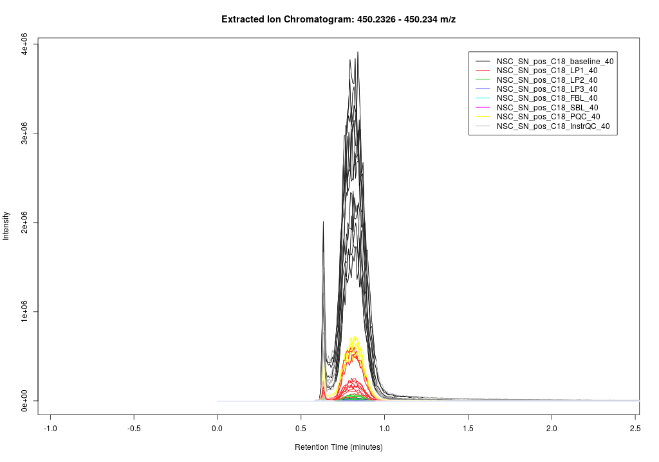


**K**
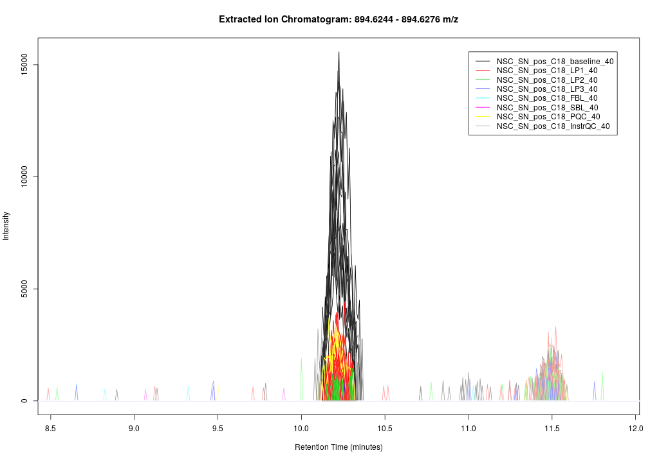
**L**
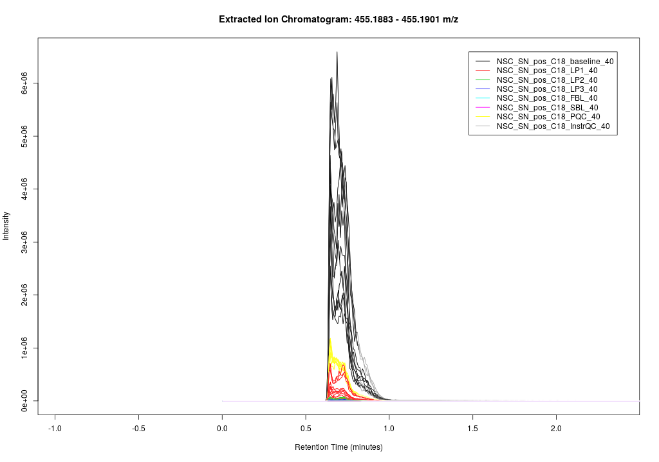
**M**
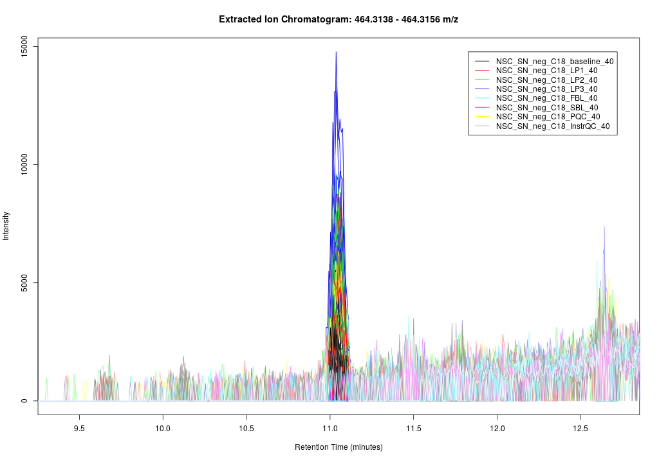
**N**
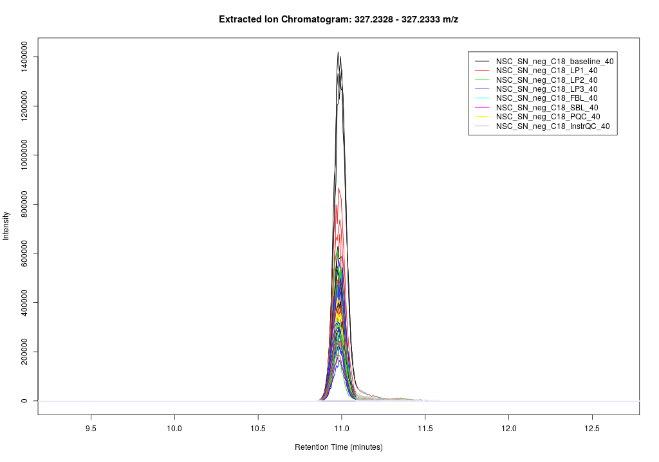


**O**
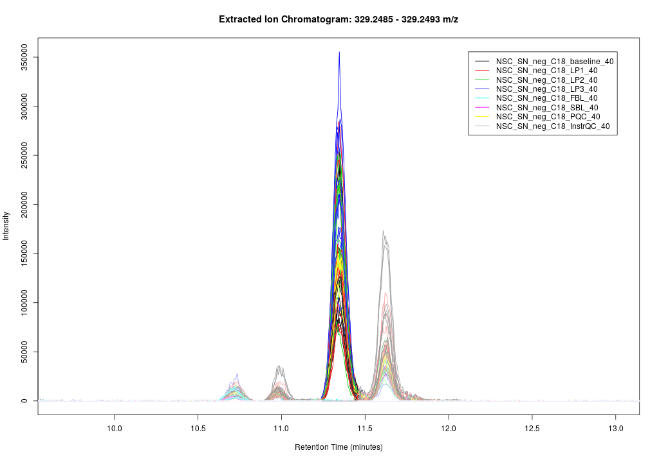
**P**
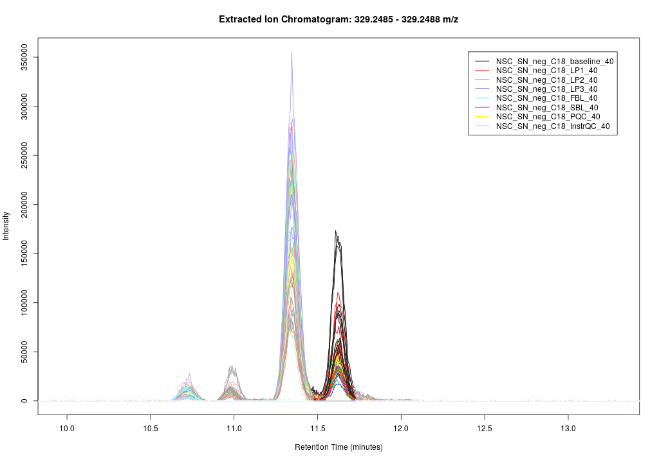


**Sup. Fig. S14.** Extracted ion chromatograms of selected metabolites and lipids presented in Sup. Table S2 extracted with C18 probes.
